# Supplementary material for: NADH-bound AIF activates the mitochondrial CHCHD4/MIA40 chaperone by a substrate-mimicry mechanism
Source: EMBO J. 2025 Jan 13;44(4):1220–48. doi: 10.1038/s44318-024-00360-6 (PMC11832770; doi:10.1038/s44318-024-00360-6)
Supplement: Supplementary file 1 — Appendix [file 44318_2024_360_MOESM1_ESM.pdf]

Appendix for

NADH-bound AIF activates the mitochondrial CHCHD4/MIA40 chaperone by a substrate mimicry mechanism

Chris A. Brosey, Runze Shen, John A. Tainer

Appendix Figures **Page**

Appendix Figure S1. AIF allosterically switches in response to NADH and binds CHCHD4 2

Appendix Figure S2. The AIF-CHCHD4 chimera mimics the allosteric state of the AIF-CTC dimer ..4

Appendix Figure S3. BilboMD minimum ensemble fitting of AIF-CHCHD4 chimera SAXS data........6

Appendix Figure S4. Full-length metazoan and fungal Mia40 AlphaFold models 7

Appendix Figure S5. The AIF-interaction motif mimics CHCHD4 substrates and contacts the CHCHD4 catalytic domain 8

Appendix Figure S6. The AIF-interaction motif regulates CHCHD4 chaperone activity 9

Appendix Figure S7. The AIF-interaction motif hinders CHCHD4 intermolecular disulfide bonds......10

Appendix Figure S8. SDS-PAGE analysis of purified protein reagents 11

Appendix Tables

Appendix Table S1. SAXS collection and analysis parameters 12

Appendix Table S2. X-ray data collection and refinement statistics for AIF-W196A-CHCHD4-N45 Chimera 15

Appendix Table S3. Average *B*-factors for individual AIF and CHCHD4 domains 16

Appendix References 17

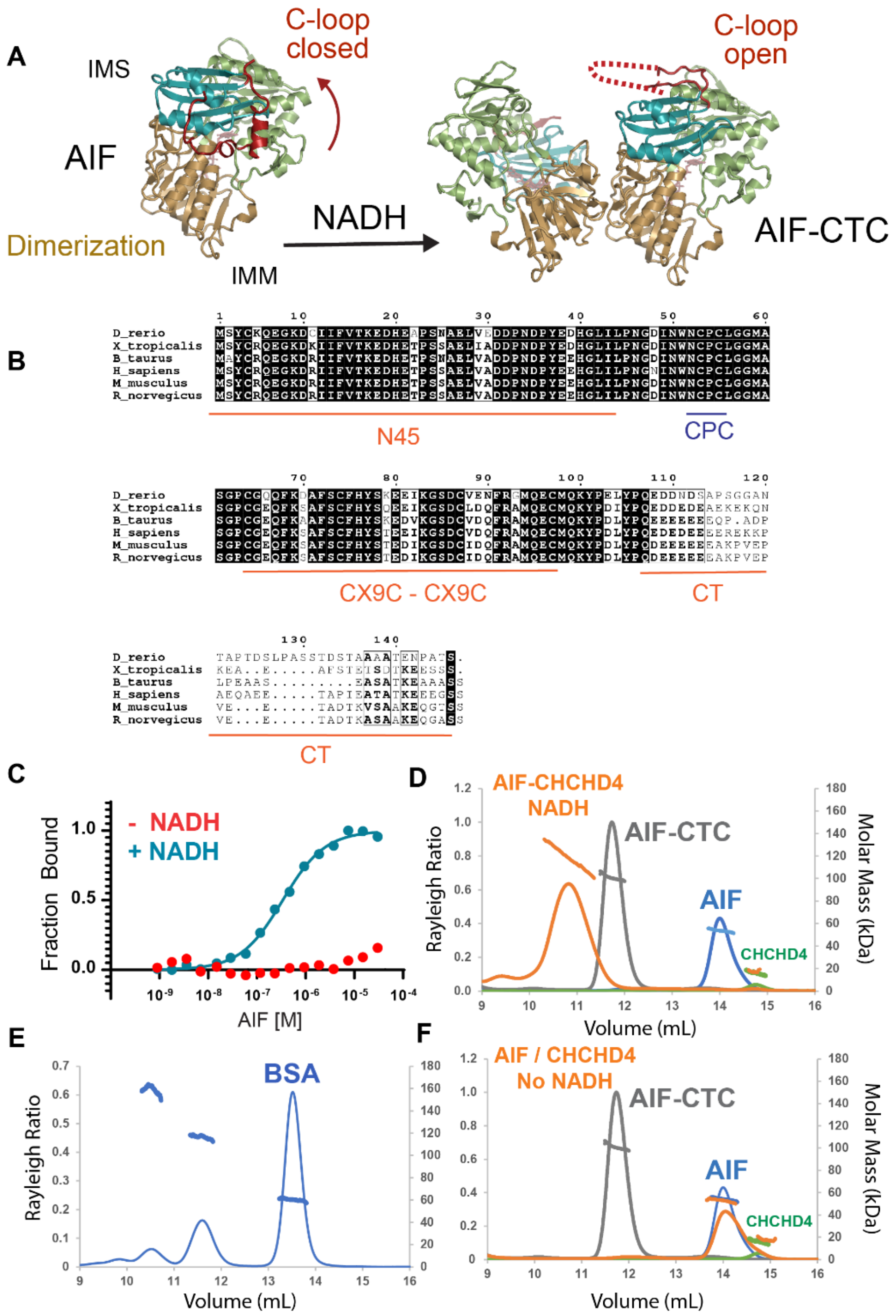

Appendix Figure S1. AIF allosterically switches in response to NADH and binds CHCHD4. (A) NADH binding, FAD reduction and formation of a charge-transfer complex (CTC) allosterically stimulates dimerization of AIF's NADH-binding domain (gold) and release of the surface C-loop (red). **PDB: 4BV6** (AIF monomer), **4BUR** (AIF dimer). **(B)** Multi-sequence alignment of metazoan CHCHD4 homologs. **(C)** MST demonstrates NADH-dependent binding between AIF and Atto488-CHCHD4. Each curve

represents the average of 3 thermophoresis scans of a representative binding titration into Atto488-CHCHD4. **(D)** SEC-MALS analysis captures the AIF-CHCHD4 complex stimulated by NADH. **(E)** SEC-MALS BSA standard. **(f)** SEC-MALS analysis of AIF and CHCHD4 co-incubated without NADH. SEC-MALS molar masses and polydispersity values are reported in **Table EV1.**

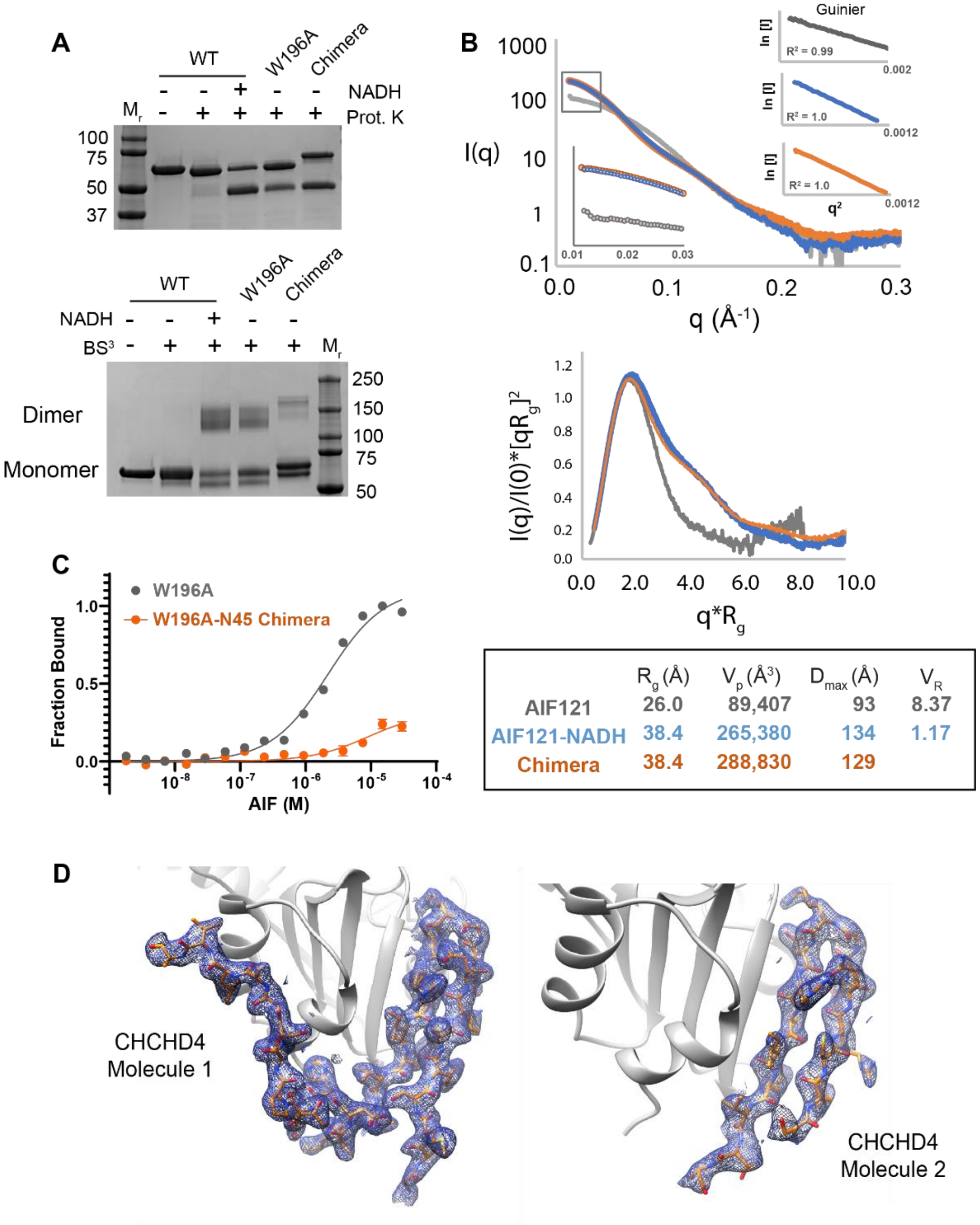

Appendix Figure S2. The AIF-CHCHD4 chimera mimics the allosteric state of the AIF-CTC dimer. (A) Without addition of NADH, the AIF-W196A-CHCHD4-N45 chimera assumes the allosteric hallmarks of the NADH-induced AIF-CTC, including C-loop displacement (upper panel) detected by Proteinase K limited proteolysis and dimerization (lower panel) detected by BS^3 amine cross-linking. **(B)** SAXS analysis of the AIF-W196A-CHCHD4-N45 chimera. Superimposed scattering profiles I(q) and Kratky transformations support structural similarity between the AIF-CHCHD4 chimera (orange) and the AIF

(121-613)-NADH dimer (SIMPLE SCATTERING entry **XSUTRZQL**) (blue) (Brosey, Ho et al., 2016, Murray, Shin et al., 2023). I(q) plot insets display a zoomed view of the low-q region (bottom) and linear, aggregation-free Guinier transformations (right). The table reports values of radius-of-gyration (R_g), Porod volume (V_p), and maximum dimension (D_{max}). Volatility-of-ratio (V_R) similarity values are calculated with reference to the AIF-CHCHD4 chimera scattering curve and demonstrate high similarity between the chimera and NADH-activated AIF (121-613) dimer. **(C)** MST titrations confirm reduced binding between Atto488-CHCHD4 and AIF-W196A when the CHCHD4 (1-45) C-terminal fusion is present. Each curve represents the average of 3 thermophoresis scans of a representative binding titration into Atto488-CHCHD4. **(D)** CHCHD4-N45 $2F_o$-F_c electron density maps contoured to 1.0σ and cut at 1.5 Å.

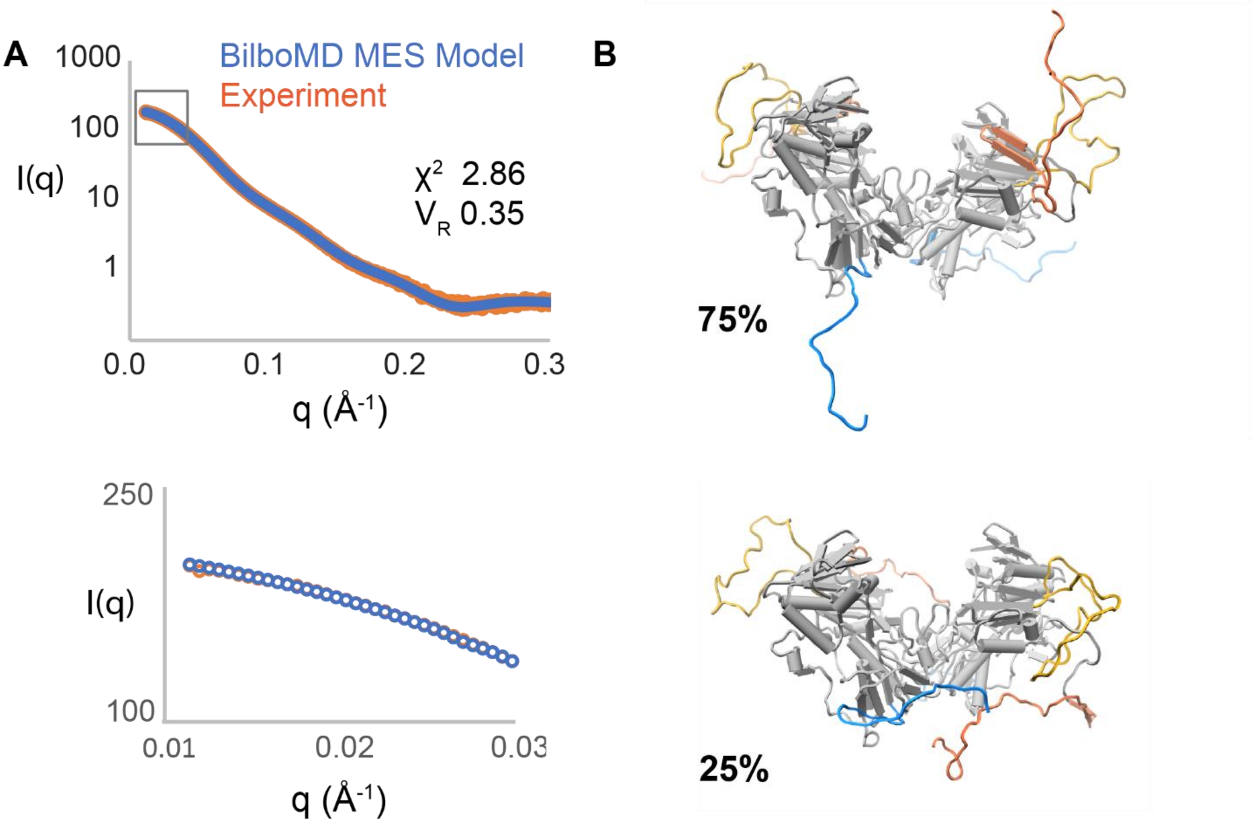

Appendix Figure S3. BilboMD minimum ensemble fitting of AIF-CHCHD4 chimera SAXS data. (A) Simulated X-ray scattering curve from weighted superposition of models identified by BilboMD minimum ensemble search (MES) (blue) overlayed with the experimental X-ray scattering curve from the AIF-CHCHD4 chimera. The lower panel shows a zoomed view of the boxed high-q region. **(B)** AIF-CHCHD4 chimera BilboMD models identified by MES. The N-terminus is highlighted in blue, C-loops in gold, and CHCHD4-N45 with C-terminus in orange.

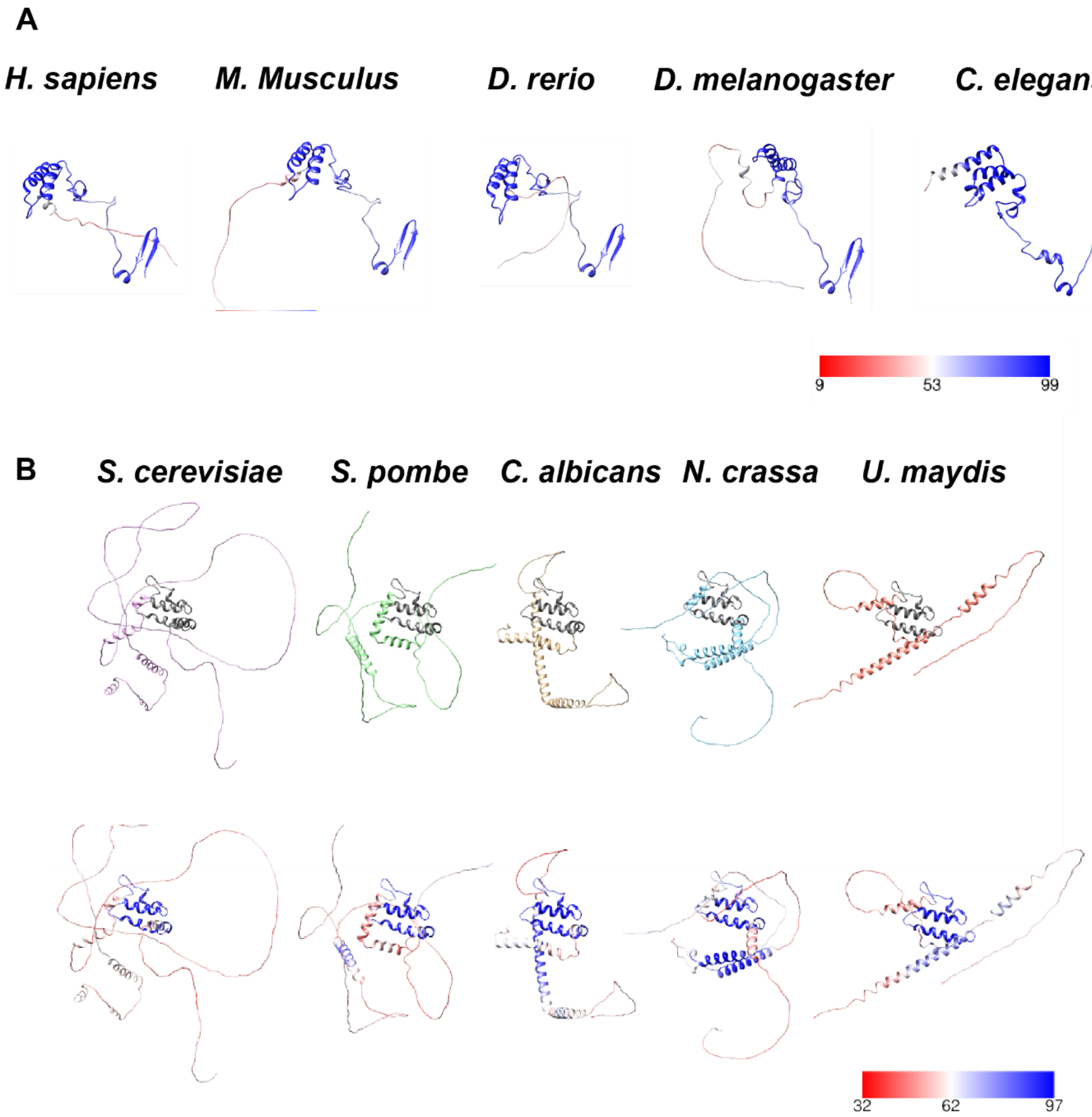

Appendix Figure S4. Full-length metazoan and fungal Mia40 AlphaFold models. (A) Metazoan CHCHD4 AlphaFold models colored by pLDDT score. **(B)** Full-length fungal AlphaFold models demonstrating variation in N-terminal domains (upper panel, central domains colored gray) and colored by pLDDT score (lower panel).

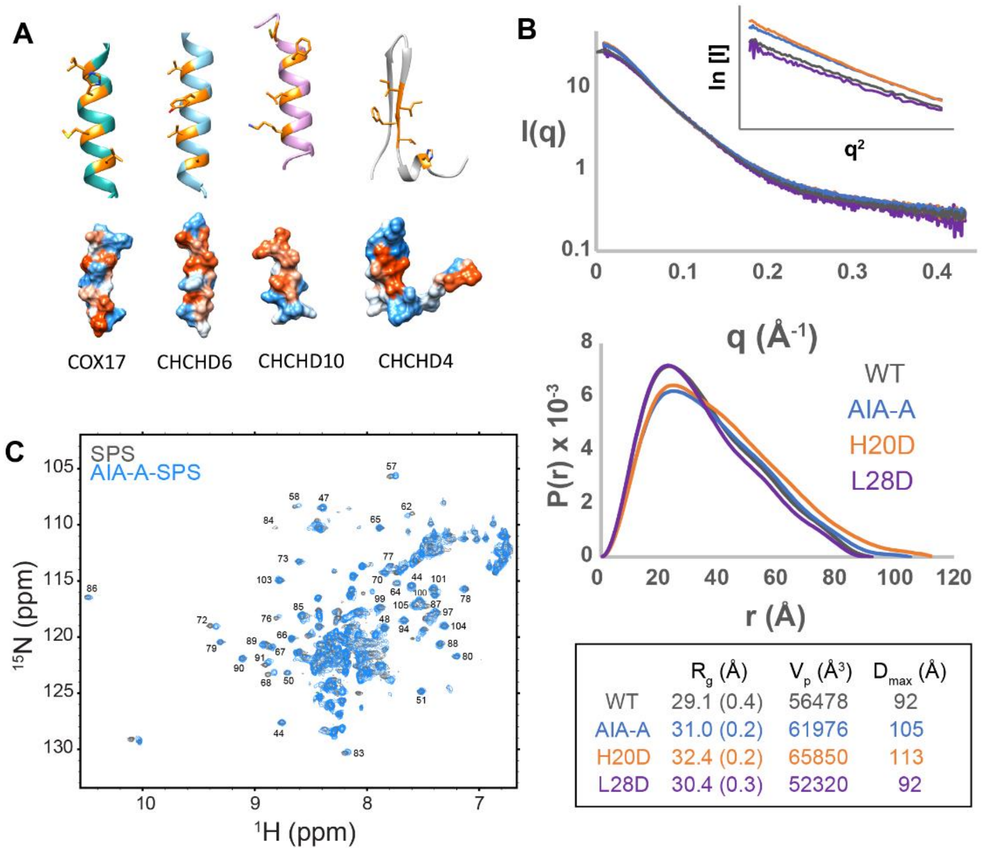

Appendix Figure S5. The AIF-interaction motif mimics CHCHD4 substrates and contacts the CHCHD4 catalytic domain. (A) Helical CHCHD4 recognition motifs (orange residues) and Kyle-Doolittle hydrophobic surfaces from import substrates COX17 (**PDB: 2L0Y**), CHCHD6 (AlphaFold), and CHCHD10 (AlphaFold) relative to CHCHD4's AIF-interaction domain. **(B)** SAXS analysis of CHCHD4 mutants. Scattering intensity profiles I(q) with Guinier plots (inset) and P(r) paired-distance distributions. **(C)** Superimposed ^{1}H-^{15}N-HSQC spectra from full-length, inactive CHCHD4 (SPS, gray) and CHCHD4 defective for AIF-interaction (AIA-A-SPS, blue). Assignments are transferred from wild-type CHCHD4 (**BMRB: 17646**) (Banci, Bertini et al., 2009).

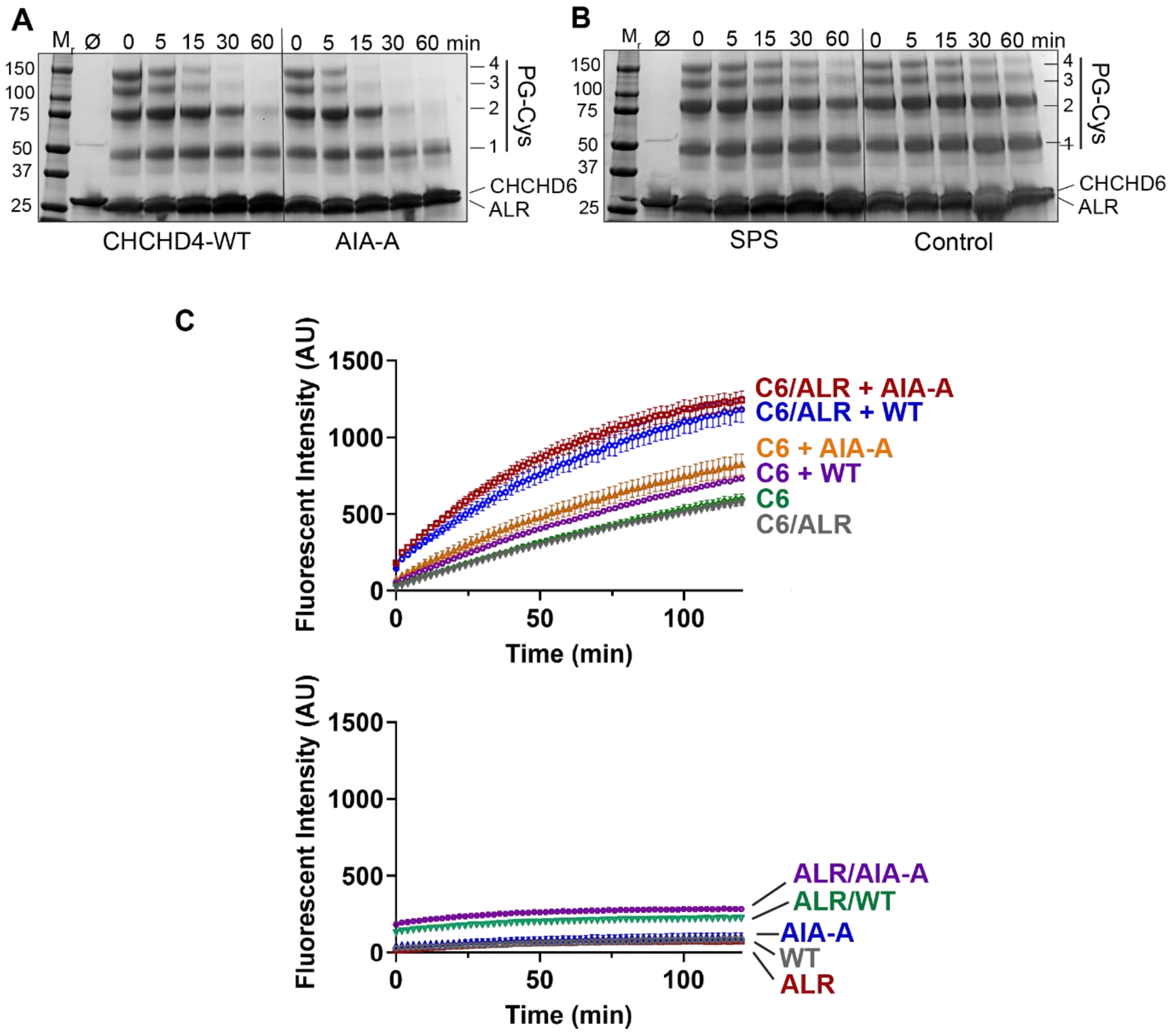

Appendix Figure S6. The AIF-interaction motif regulates CHCHD4 chaperone activity. (A) Time-course of *in vitro* chaperone activity for wild-type and AIA-A CHCHD4 for unfolded CHCHD6 monitored by PEG-MEM labeling and SDS-PAGE. **(B)** Time-course of *in vitro* chaperone activity for CHCHD4-SPS and the control reaction (no CHCHD4) for unfolded CHCHD6 monitored by PEG-MEM labeling and SDS-PAGE. **(C)** Time-resolved Amplex Red fluorescent detection of hydrogen peroxide from CHCHD4 disulfide relay activity with all controls. Traces are the average of 3 technical replicates with error bars representing standard deviations. Results are representative of 3 independent experiments. The top panel is reproduced from **Figure 5B**. C6, CHCHD6; WT, CHCHD4-WT; AIA-A, CHCHD4-AIA-A.

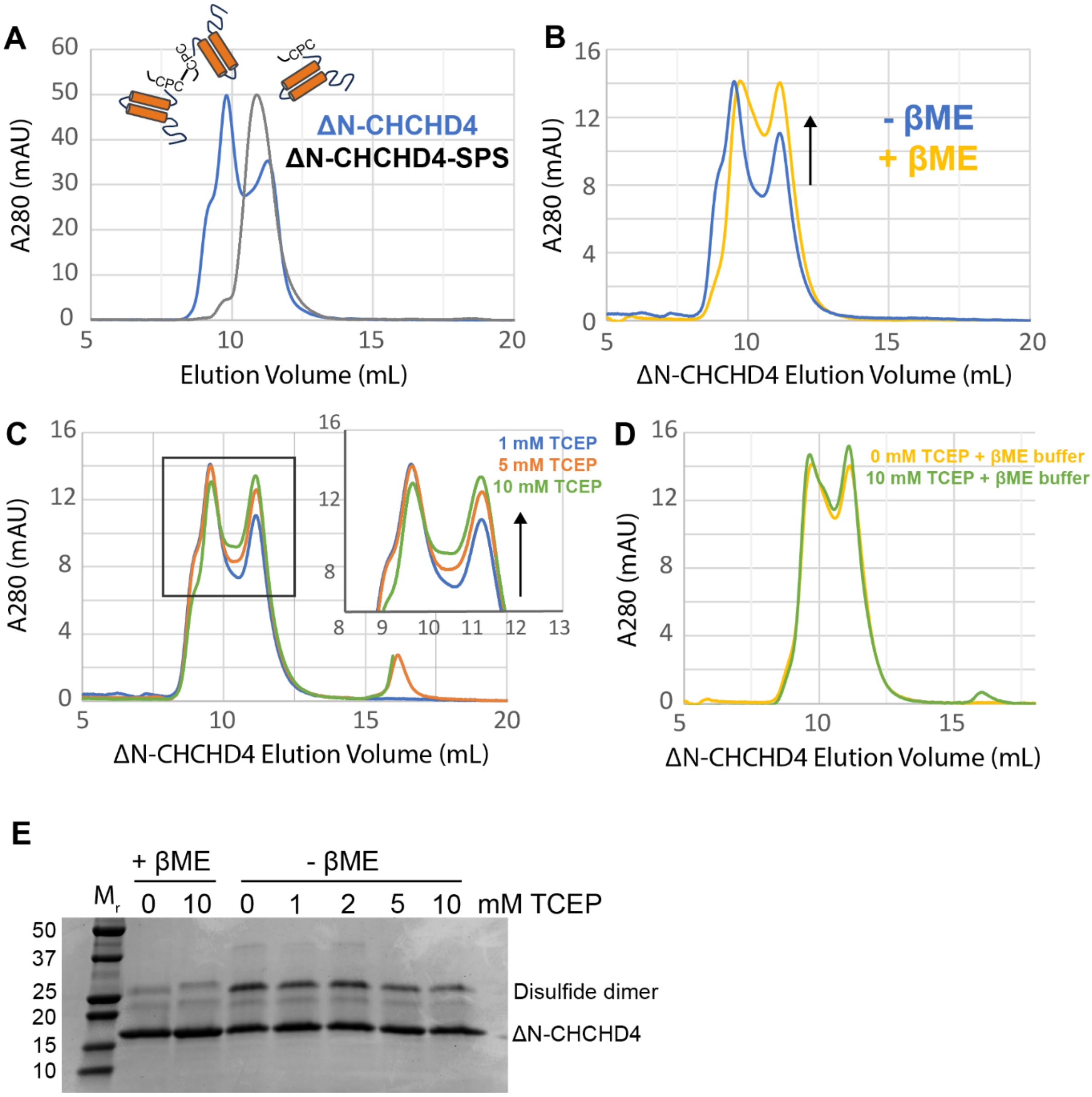

Appendix Figure S7. The AIF-interaction motif hinders CHCHD4 intermolecular disulfide bonds. **(A)** Size-exclusion chromatograms of active and inactive N-terminally truncated CHCHD4. **(B)** Overlay of SEC curves from ΔN-CHCHD4 eluted in buffer without and with 5 mM βME. **(C)** SEC analysis of ΔN-CHCHD4 pre-treated with TCEP. The curve at 0 mM TCEP is reproduced from panel B. **(D)** SEC analysis of ΔN-CHCHD4 pre-treated with TCEP and eluted with and without βME. The curve at 0 mM TCEP is reproduced from panel B. **(E)** Non-reducing and reducing SDS-PAGE of TCEP-treated ΔN-CHCHD4.

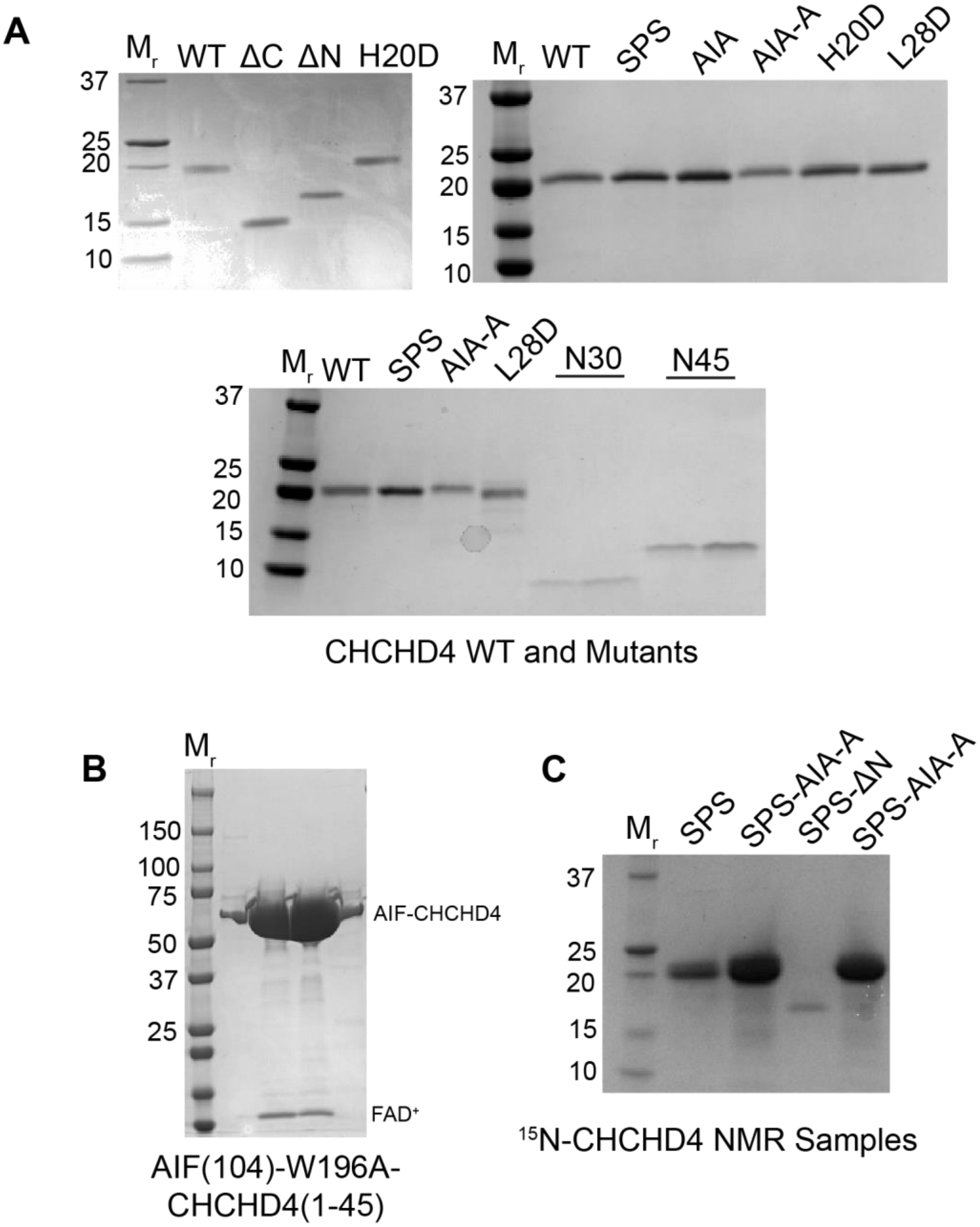

Appendix Figure S8. SDS-PAGE analysis of purified protein reagents. (A) Wild-type CHCHD4 and mutants: ΔC (residues 1-105), ΔN (residues 45-143), SPS (C53S/C55S), AIA (I12A/F14A), AIA-A (I12A/F14A/H20A), N30 (residues 1-30), N45 (residues 1-45). **(B)** AIF-CHCHD4 fusion: AIF-W196A (residues 104-613) – SGSGPGSGS – CHCHD4 (residues 1-45). **(C)** ^{15}N-enriched wild-type and mutant CHCHD4 NMR samples.

Appendix Table S1. SAXS Collection and Analysis Parameters

Sample Details	AIF-CHCHD4-Chimera
Organism	*H. sapiens*
Source	*E. coli* expressed
UniProt ID (residues in construct)	O95831 (AIF 104-613, W196A), Q8N4Q1 (CHCHD4 1-45)
Extinction coefficient (FAD) (A_{450}, M^{-1} cm^{-1})	13,400 (Churbanova & Sevrioukova, 2008)
$\bar{v}$ from chemical composition (cm^3 g^{-1})	0.737
Particle contrast from sequence and solvent components, $\Delta\bar{\rho}$ ($\rho_{protein} - \rho_{solvent}$; 10^{10} cm^{-2})	2.84 (12.31 – 9.46)
M_r from chemical composition (Da)	62,191
Solvent (buffer for subtraction taken from preparatory SEC flowthrough prior to elution of protein)	25 mM HEPES, pH 7.5, 150 mM NaCl, 5 mM βME
HT-SAXS samples concentrations	4.9 mg/mL
HT-SAXS sample volume	30 µL

Sample Details	CHCHD4 WT	CHCHD4 AIA-A	CHCHD4 H20D	CHCHD4 L28D
Organism	*H. sapiens*	*H. sapiens*	*H. sapiens*	*H. sapiens*
Source	*E. coli* expressed	*E. coli* expressed	*E. coli* expressed	*E. coli* expressed
UniProt ID (residues in construct)	Q8N4Q1	Q8N4Q1 (I12A/F14A/H20A)	Q8N4Q1 (H20D)	Q8N4Q1 (L28D)
Extinction coefficient (A_{280}, M^{-1} cm^{-1})	13,110 (Gasteiger, Hoogland et al., 2005)	13,110 (Gasteiger et al., 2005)	13,110 (Gasteiger et al., 2005)	13,110 (Gasteiger et al., 2005)
$\bar{v}$ from chemical composition (cm^3 g^{-1})	0.710	0.709	0.710	0.708
Particle contrast from sequence and solvent components, $\Delta\bar{\rho}$ ($\rho_{protein} - \rho_{solvent}$; 10^{10} cm^{-2})	3.23 (12.69 – 9.46)	3.24 (12.71 – 9.46)	3.23 (12.70 – 9.46)	3.25 (12.72 – 9.46)
M_r from chemical composition (Da)	15,996	15,996	15,996	15,996
Solvent (buffer for subtraction taken from preparatory SEC flowthrough prior to sample elution or from flowthrough of concentrated samples)	25 mM HEPES, pH 7.5, 150 mM NaCl, 2 mM TCEP	25 mM HEPES, pH 7.5, 150 mM NaCl, 2 mM TCEP	25 mM HEPES, pH 7.5, 150 mM NaCl, 2 mM TCEP	25 mM HEPES, pH 7.5, 150 mM NaCl, 2 mM TCEP
HT-SAXS samples concentrations	5.2 mg/mL	7.3 mg/mL	3 mg/mL	6 mg/mL
HT-SAXS sample volume	30 µL	30 µL	30 µL	30 µL

Appendix Table S1. SAXS Collection and Analysis Parameters (continued)

SAXS Data-Collection Parameters

Instrument/data processing	Advanced Light Source (ALS) SIBYLS SAXS beamline (12.3.1) with Dectris Pilatus3 2M Detector (Classen, Hura et al., 2013)
Wavelength (Å)	1.27 (AIF-CHCHD4 chimera), 1.23 (CHCHD4)
Beam size (µm)	Converging beam, 500x2000 at sample, 100x100 at detector
Camera length (m)	2
q measurement range ($Å^{-1}$)	0.01-0.39 (AIF-CHCHD4-Chimera), 0.01-0.43 (CHCHD4)
Absolute scaling method	On a detector scale
Normalization	To transmitted intensity by beam-stop counter
Monitoring for radiation damage	Data and R_g frame-by-frame comparison
Exposure time	0.5 s for 15 s (AIF-CHCHD4-Chimera) or 0.3 s for 10 s (CHCHD4)
Sample configuration	Samples were loaded by a multichannel Tecan Evo 100 liquid-handling robot into needle tips containing mica windows. Effective sample path length 1.5 mm.
Sample temperature (°C)	10

SAXS Analysis Software

SAXS data reduction	Advanced Light Source (ALS) SIBYLS SAXS beamline (12.3.1) with Dectris Pilatus3 2M Detector (Classen et al., 2013)<br>SAXS FrameSlice (https://sibyls.als.lbl.gov/ran/instructions)
Extinction coefficient estimate	*ProtParam* (Gasteiger et al., 2005)
Calculation of $\bar{v}$ and $\Delta\bar{\rho}$	MULCh v 1.1.1 (Whitten, Cai et al., 2008) (https://smb-research.smb.usyd.edu.au/NCVWeb/index.jsp)
First-order analyses, I(0), R_g	ScÅtter 3.0 (https://bl1231.als.lbl.gov/scatter/), Primus/qt ATSAS 3.0.1 (Franke, Petoukhov et al., 2017)
Graph visual display	Microsoft Excel (version 2303)

**SIMPLE SCATTERING ID**	XS6ARGD0 (AIF-CHCHD4-Chimera)<br>XS4FJZBK (CHCHD4 wild-type and mutants)
**SASBDB ID**	SDSDV47 (AIF-CHCHD4 chimera)<br>SASDVY6 (CHCHD4 wild-type)<br>SASDVZ6 (CHCHD4 F12A/I14A/H20A, 'AIA-A')<br>SASDV27 (CHCHD4 H20D)<br>SASDV37 (CHCHD4 L28D)

Appendix Table S1. SAXS Collection and Analysis Parameters (continued)

Structural Parameters	AIF-CHCHD4-Chimera
Guinier Analysis	
I(0)	197 (0.21)
R_g (Å)	38.4 (0.23)
q_{min} (Å^{-1})	0.0125
qR_g max (q_{min} = 0.0125 Å^{-1})	1.30
Coefficient of correlation, R^2	0.99
Mass from Porod invariant (Da) (Rambo & Tainer, 2013)	110,790
Real-space Analysis	
I(0)	193 (1.27)
R_g (Å)	38.5 (0.22)
D_{max} (Å)	129
q range (Å^{-1})	0.0276 - 0.3843
χ^2	2.76
Porod volume, V_p (Å^3)	288,830
Porod coefficient, P_x	3.90 (0.05)

Structural Parameters	CHCHD4 WT	CHCHD4 AIA-A	CHCHD4 H20D	CHCHD4 L28D
Guinier Analysis				
I(0)	29.6 (0.08)	48.9 (0.07)	36.5 (0.05)	34.2 (0.10)
R_g (Å)	29.1 (0.37)	30.95 (0.24)	32.42 (0.24)	28.31 (0.42)
q_{min} (Å^{-1})	0.0094	0.0094	0.0094	0.0094
qR_g max (q_{min} = 0.0144 Å^{-1})	1.29	1.28	1.29	1.30
Coefficient of correlation, R^2	0.99	1.00	1.00	0.98
Mass from Porod invariant (Da) (Rambo & Tainer, 2013)	17,400	19,800	21,200	17,200
Real-space Analysis				
I(0)	23.7 (0.26)	39.2 (0.39)	29.1(0.30)	27.5 (0.97)
R_g (Å)	28.09 (0.17)	29.85 (0.17)	31.74 (0.25)	27.13 (0.34)
D_{max} (Å)	92	105	113	92
q range (Å^{-1})	0.0241 – 0.28756	0.0241 - 0.3128	0.0241 - 0.3128	0.0241 - 0.3889
χ^2	0.07	0.05	0.03	0.32
Porod volume, V_p (Å^3)	56,478	61,976	65,850	52,320
Porod coefficient, P_x	2.67 (0.02)	2.34 (0.02)	2.38 (0.02)	2.72 (0.04)

Appendix Table S2. X-ray data collection and refinement statistics for AIF-W196A- CHCHD4-N45 Chimera.

	AIF-W196A-CHCHD4-N45
**Data collection**	
Space group	$P2_12_12_1$
Cell dimensions	
a, *b*, *c* (Å)	68.61 109.20 174.63
α, β, γ (°)	90, 90, 90
Resolution (Å)	92.59 - 2.3 (2.38 - 2.3)
R_{merge}	0.142 (1.529)
$I / \sigma I$	13.36 (1.80)
Completeness (%)	99.96 (99.98)
Redundancy	13.3 (13.8)
**Refinement**	
Resolution (Å)	2.30
No. reflections	59106 (5832)
R_{work} / R_{free}	18.1% / 21.2%
No. atoms	7566
Protein	7064
Ligand/ion	114
Water	388
B-factors	52.66
Protein	52.99
Ligand/ion	40.89
Water	50.26
R.m.s. deviations	
Bond lengths (Å)	0.002
Bond angles (°)	0.55

*Values in parentheses are for highest-resolution shell.

Appendix Table S3. Average *B*-factors for individual AIF and CHCHD4 domains

	**Average B-factor**	**Median B-factor**
AIF Molecule 1	50.89	46
AIF Molecule 2	54.35	52
CHCHD4-N45 Molecule 1	51.91	46
CHCHD4-N45 Molecule 2	73.67	70

Appendix References

Banci L, Bertini I, Cefaro C, Ciofi-Baffoni S, Gallo A, Martinelli M, Sideris DP, Katrakili N, Tokatlidis K (2009) MIA40 is an oxidoreductase that catalyzes oxidative protein folding in mitochondria. Nature Structural & Molecular Biology 16: 198-206

Brosey CA, Ho C, Long WZ, Singh S, Burnett K, Hura GL, Nix JC, Bowman GR, Ellenberger T, Tainer JA (2016) Defining NADH-Driven Allostery Regulating Apoptosis-Inducing Factor. Structure 24: 2067-2079

Churbanova IY, Sevrioukova IF (2008) Redox-dependent changes in molecular properties of mitochondrial apoptosis-inducing factor. J Biol Chem 283: 5622-31

Classen S, Hura GL, Holton JM, Rambo RP, Rodic I, McGuire PJ, Dyer K, Hammel M, Meigs G, Frankel KA et al. (2013) Implementation and performance of SIBYLS: a dual endstation small-angle X-ray scattering and macromolecular crystallography beamline at the Advanced Light Source. Journal of Applied Crystallography 46: 1-13

Franke D, Petoukhov MV, Konarev PV, Panjkovich A, Tuukkanen A, Mertens HDT, Kikhney AG, Hajizadeh NR, Franklin JM, Jeffries CM et al. (2017) ATSAS 2.8: a comprehensive data analysis suite for small-angle scattering from macromolecular solutions. J Appl Crystallogr 50: 1212-1225

Gasteiger E, Hoogland C, Gattiker A, Duvaud Se, Wilkins MR, Appel RD, Bairoch A (2005) Protein Identification and Analysis Tools on the ExPASy Server. In The Proteomics Protocols Handbook, Walker JM (ed) pp 571-607. Totowa, NJ: Humana Press

Murray DT, Shin DS, Classen S, Brosey CA, Hura GL (2023) Visualizing and accessing correlated SAXS data sets with Similarity Maps and Simple Scattering web resources. Methods Enzymol 678: 411-440

Rambo RP, Tainer JA (2013) Accurate assessment of mass, models and resolution by small-angle scattering. Nature 496: 477-81

Whitten AE, Cai S, Trewhella J (2008) MULCh: modules for the analysis of small-angle neutron contrast variation data from biomolecular assemblies. Journal of Applied Crystallography 41: 222-226
