# Supplementary material for: NADH-bound AIF activates the mitochondrial CHCHD4/MIA40 chaperone by a substrate-mimicry mechanism
Source: EMBO J. 2025 Jan 13;44(4):1220–48. doi: 10.1038/s44318-024-00360-6 (PMC11832770; doi:10.1038/s44318-024-00360-6)
Supplement: Supplementary file 2 — Table EV1 [file 44318_2024_360_MOESM2_ESM.docx]

**Expanded View Tables**

**Table EV1. SEC-MALS Analysis of AIF-CHCHD4 Complexes and CHCHD4 Mutants.**

|  | **Peak 1** | | | | | **Peak 2** | | | | | | |  |  |  |  |
| --- | --- | --- | --- | --- | --- | --- | --- | --- | --- | --- | --- | --- | --- | --- | --- | --- |
|  | **Mw (kDa)** | **Uncertainty** | **Polydispersity (Mw/Mn)** | **Uncertainty** | | **Mw (kDa)** | **Uncertainty** | | **Polydispersity (Mw/Mn)** | | **Uncertainty** | |  |  |  |  |
| **BSA*** | 60.2 | 0.40% | 1 | 0.58% | | 116.3 | 0.50% | | 1 | | 0.66% | |  |  |  |  |
| **AIF** | 53.6 | 0.40% | 1 | 0.61% | |  |  | |  | |  | |  |  |  |  |
| **AIF-NADH (CTC)** | 100.7 | 0.50% | 1 | 0.75% | |  |  | |  | |  | |  |  |  |  |
| **CHCHD4 WT** | 15.9 | 1.70% | 1.006 | 2.47% | |  |  | |  | |  | |  |  |  |  |
| **CHCHD4 AIA-A** | 16.1 | 4.50% | 1.002 | 6.33% | |  |  | |  | |  | |  |  |  |  |
| **AIF-CTC / CHCHD4**** | 116.3 | 0.50% | 1.009 | 0.72% | | 18.2 | 7.30% | | 1.006 | | 9.86% | |  |  |  |  |
| **AIF-CTC / CHCHD4 AIA-A***** | 100.8 | 0.80% | 1 | 1.07% | | 16.7 | 1.40% | | 1.004 | | 2.00% | |  |  |  |  |
|  |  |  |  |  | |  |  | |  | |  | |  |  |  |  |
| * BSA monomer (Peak 1), BSA dimer (Peak 2) | | | | |  |  |  | |  | |  | |  |  |  |  |
| ** AIF-CTC-CHCHD4 complex (Peaks 1), Residual CHCHD4 (Peak 2) | | | | | | | |  | |  | |  | |  |  |  |
| ***AIF-CTC (Peak 1), Residual CHCHD4 (Peak 2) | | | | | | | |  | |  | |  | |  |  |  |
