# Supplementary figures and images for: NADH-bound AIF activates the mitochondrial CHCHD4/MIA40 chaperone by a substrate-mimicry mechanism
Source: EMBO J. 2025 Jan 13;44(4):1220–48. doi: 10.1038/s44318-024-00360-6 (PMC11832770; doi:10.1038/s44318-024-00360-6)

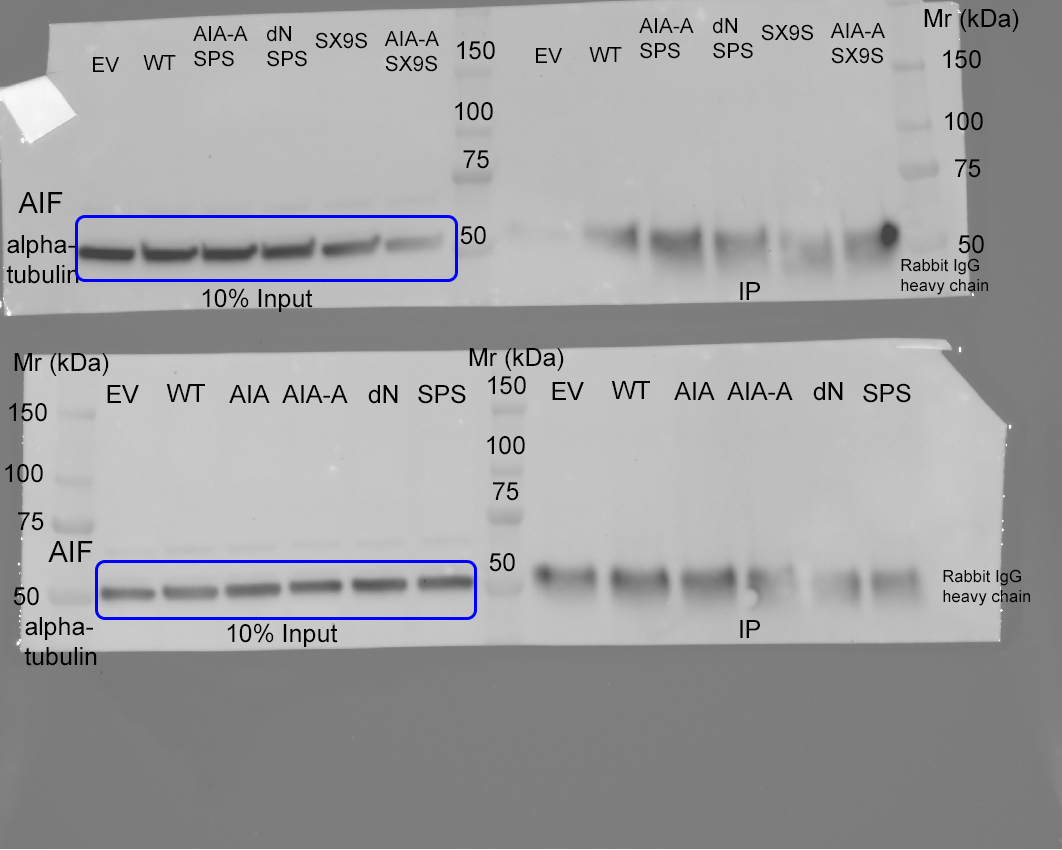

Supplement: Supplementary file 6 — Source data Fig. 2 [file 44318_2024_360_MOESM6_ESM.zip › Figure 2/2C/western a-tubulin.tif]

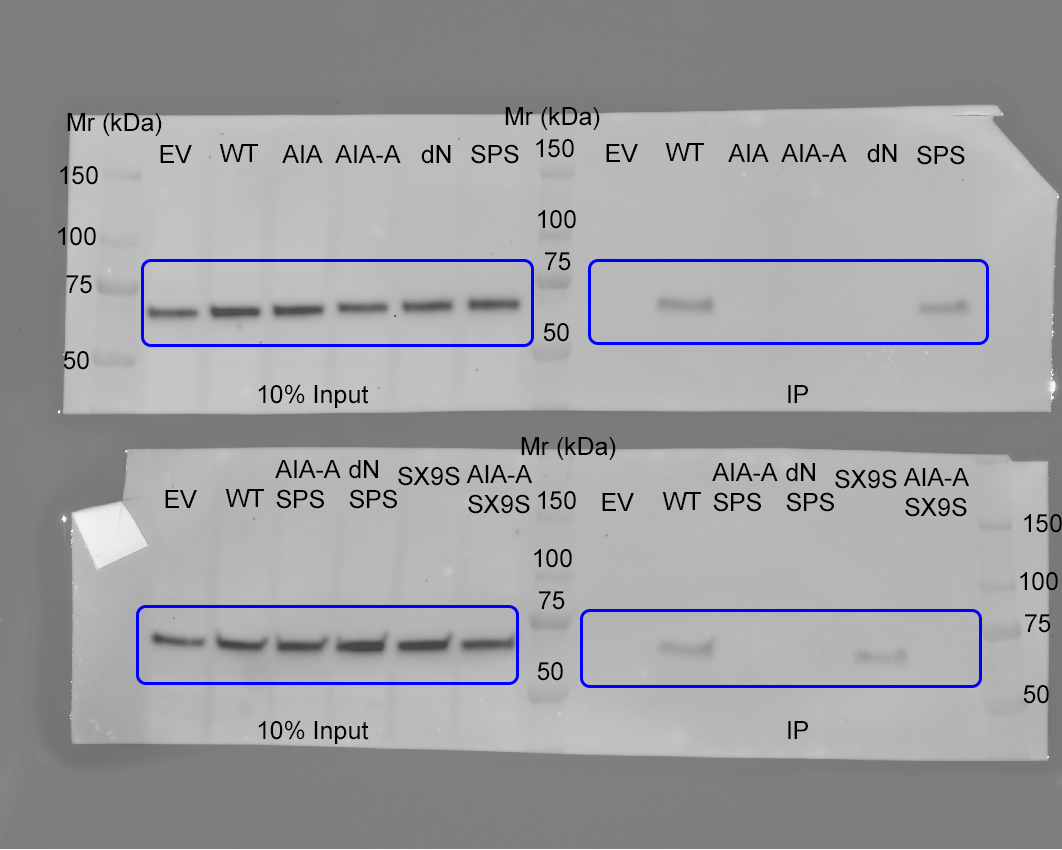

Supplement: Supplementary file 6 — Source data Fig. 2 [file 44318_2024_360_MOESM6_ESM.zip › Figure 2/2C/western AIF.tif]

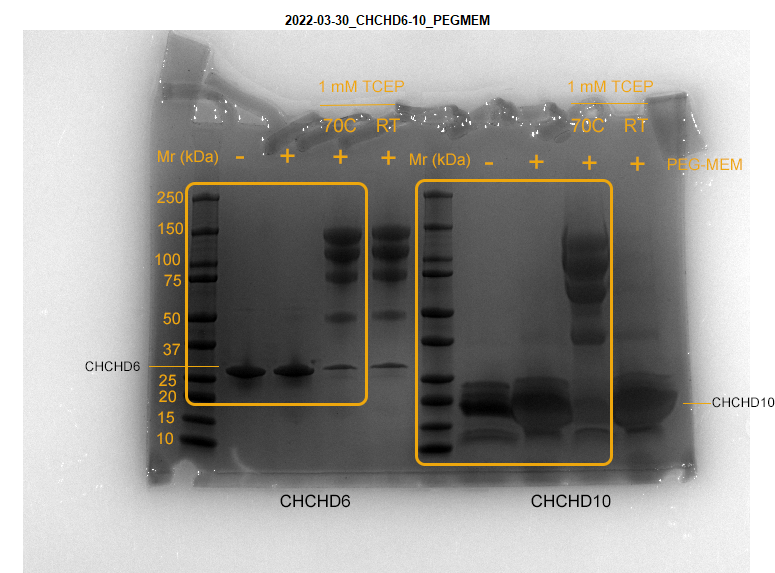

Supplement: Supplementary file 8 — Source data Fig. 4 [file 44318_2024_360_MOESM8_ESM.zip › Figure 4/4C/Figure-4C-SDS-PAGE-CHCHD6-CHCHD10.tif]

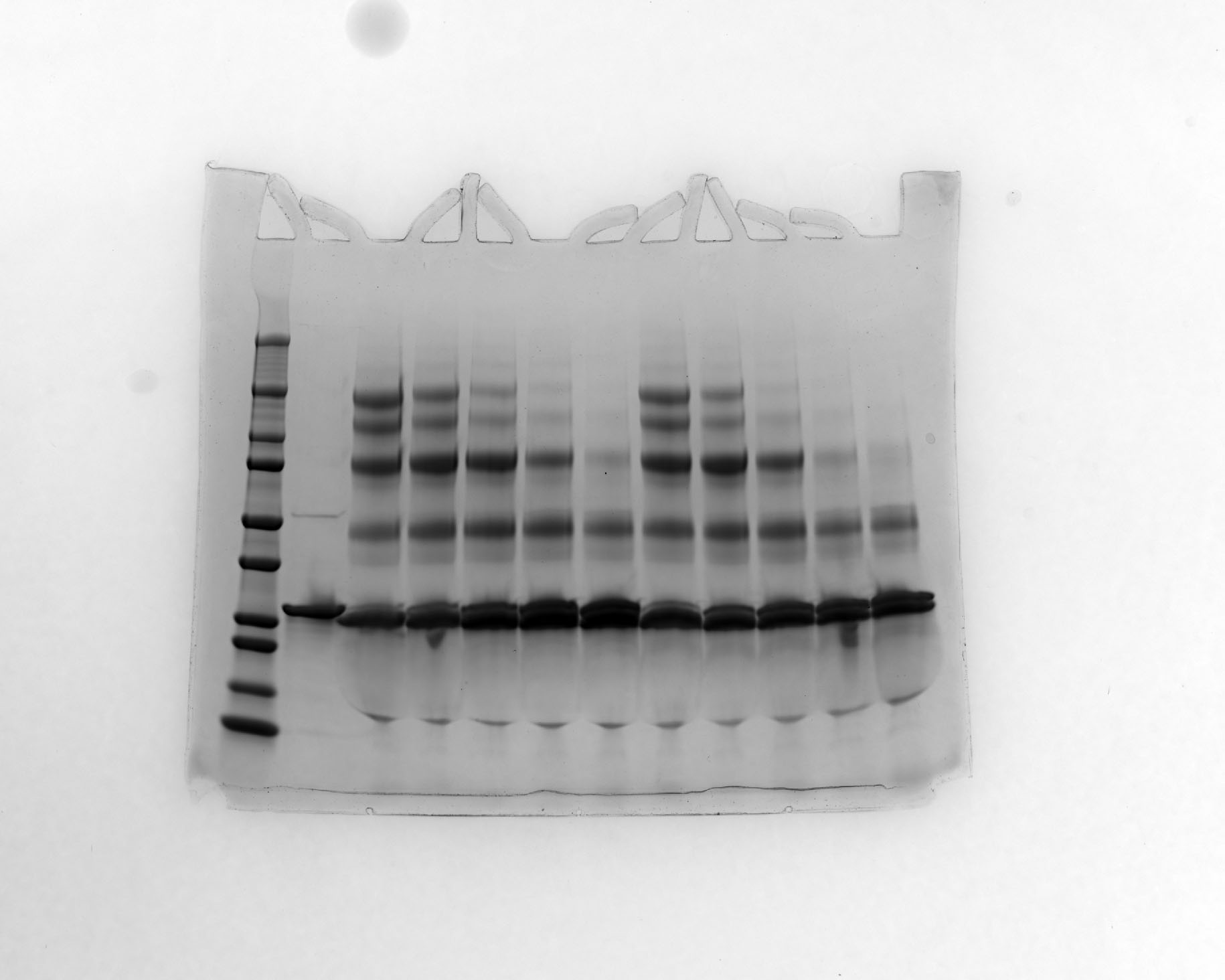

Supplement: Supplementary file 9 — Source data Fig. 5 [file 44318_2024_360_MOESM9_ESM.zip › Figure 5/5B/Figure-5B-SDS-PAGE-CHCHD4-Refolding-Timecourse.jpg]

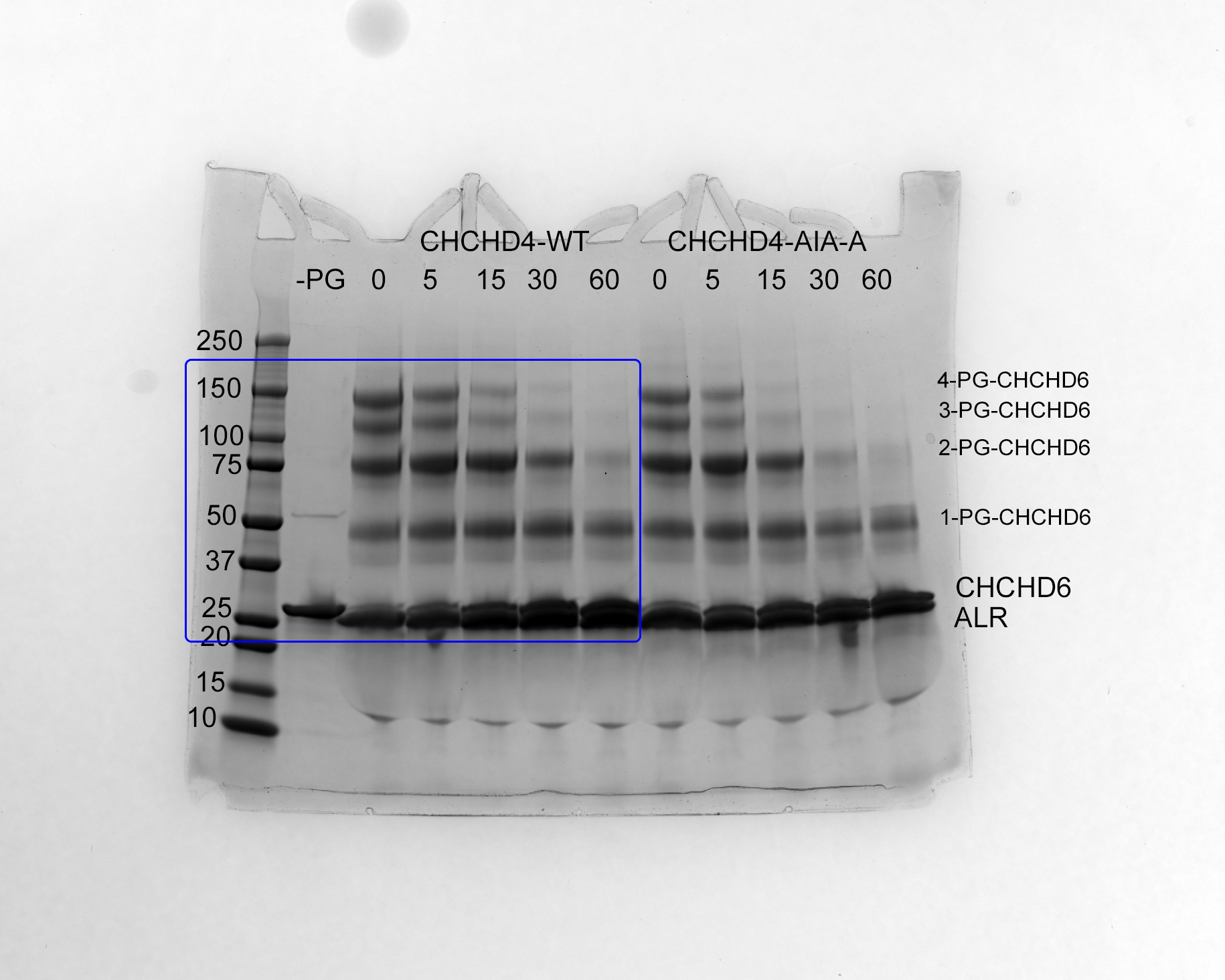

Supplement: Supplementary file 9 — Source data Fig. 5 [file 44318_2024_360_MOESM9_ESM.zip › Figure 5/5B/Figure-5B-SDS-PAGE-CHCHD4-Refolding-Timecourse.tif]

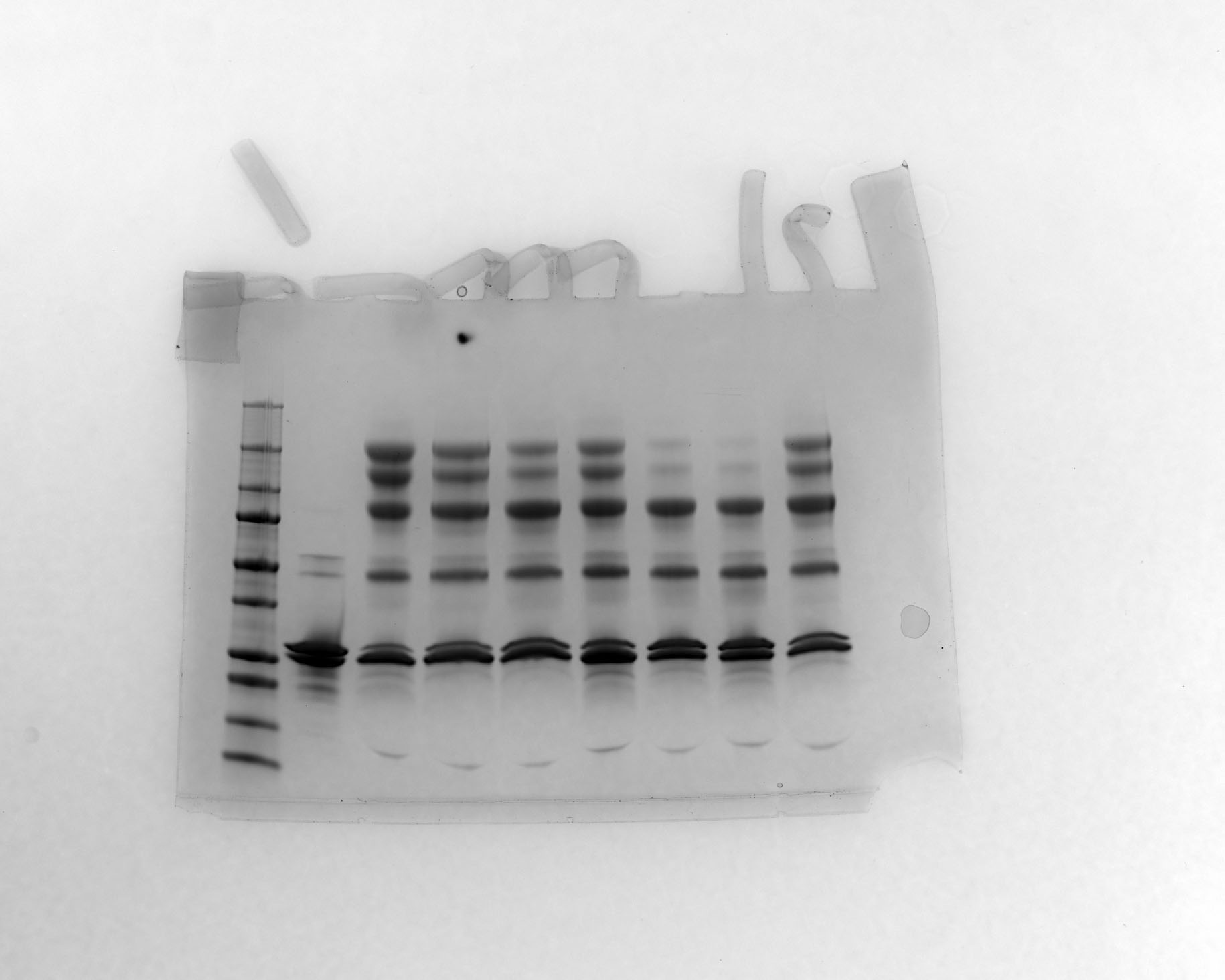

Supplement: Supplementary file 9 — Source data Fig. 5 [file 44318_2024_360_MOESM9_ESM.zip › Figure 5/5D/Figure-5D-SDS-PAGE-CHCHD4-Refolding-15min.jpg]

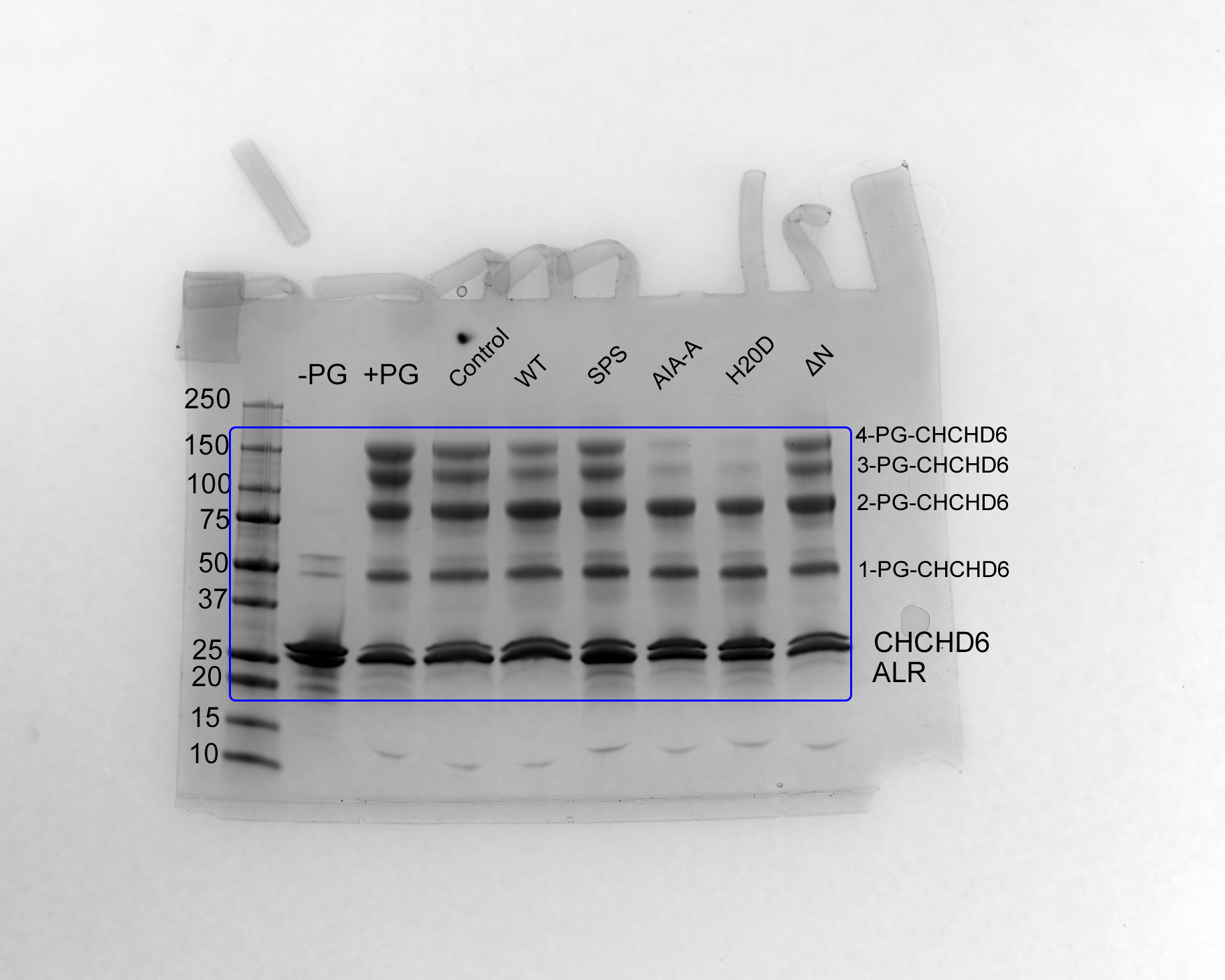

Supplement: Supplementary file 9 — Source data Fig. 5 [file 44318_2024_360_MOESM9_ESM.zip › Figure 5/5D/Figure-5D-SDS-PAGE-CHCHD4-Refolding-15min.tif]

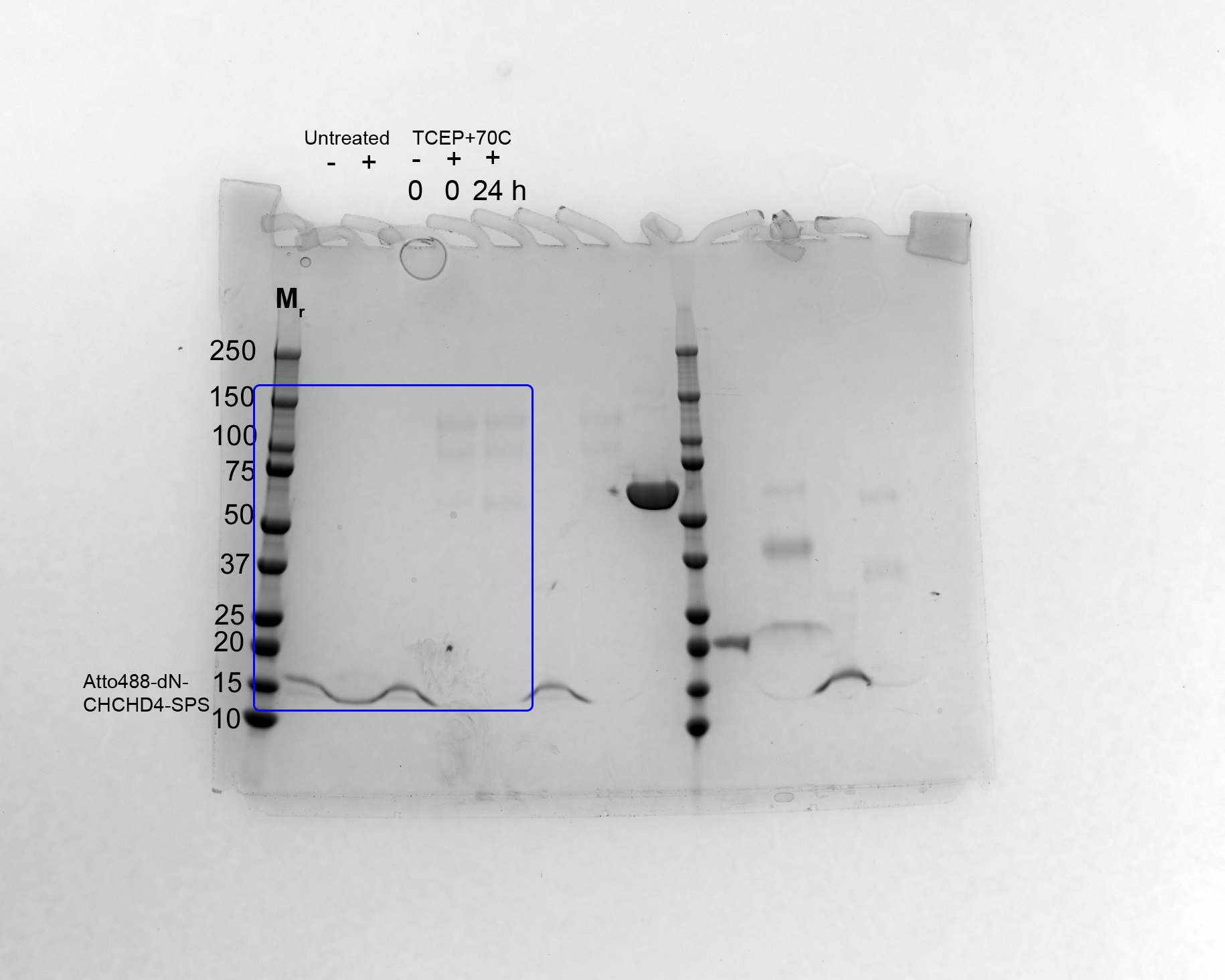

Supplement: Supplementary file 10 — Source data Fig. 6 [file 44318_2024_360_MOESM10_ESM.zip › Figure 6/6B/SDS-PAGE-Atto488-dN-CHCHD4-PEG-labeling.tif]

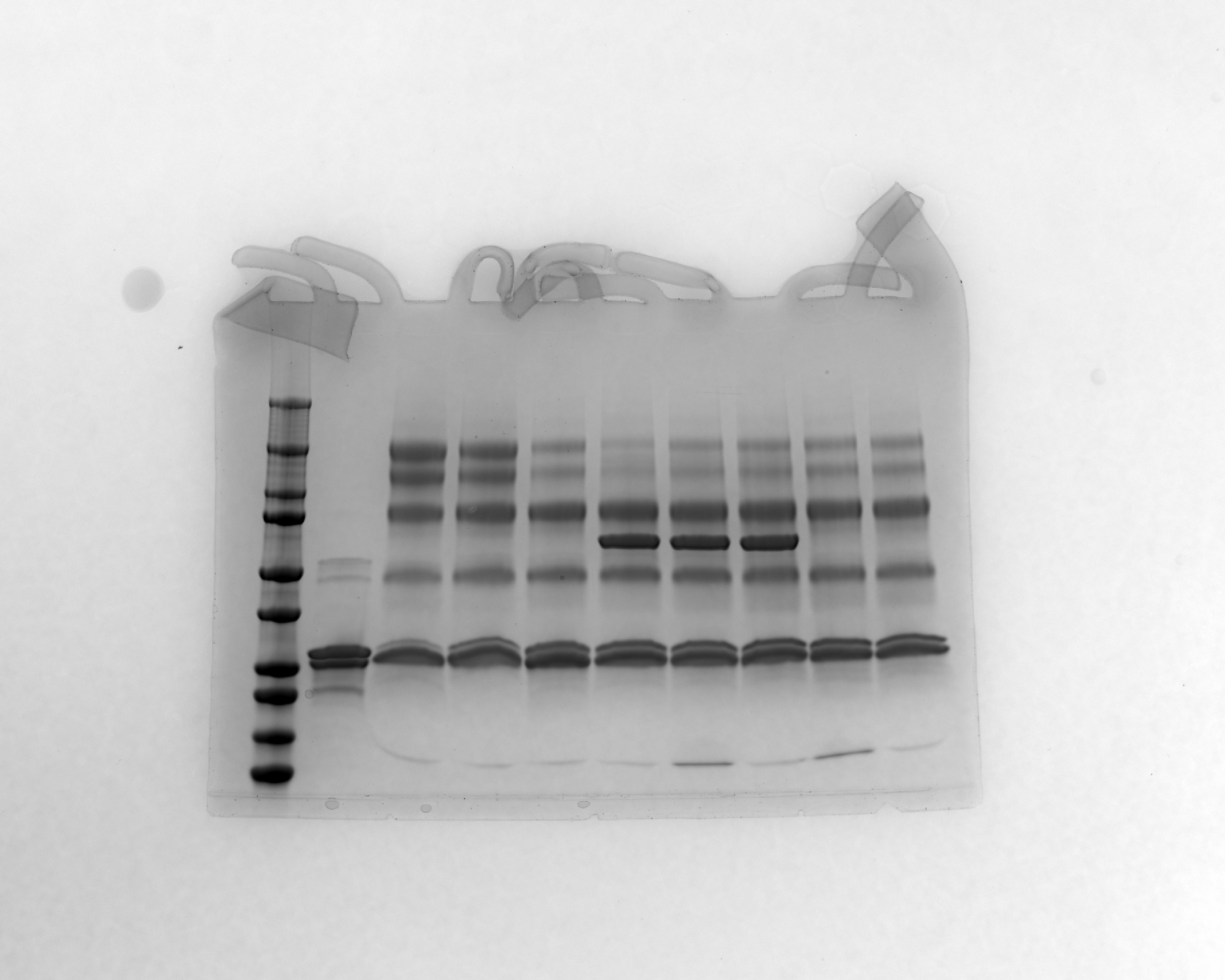

Supplement: Supplementary file 10 — Source data Fig. 6 [file 44318_2024_360_MOESM10_ESM.zip › Figure 6/6E/cabrosey 2022-09-01 10h44m16s Coomassie Blue 0.481s(Coomassie Blue).tif]

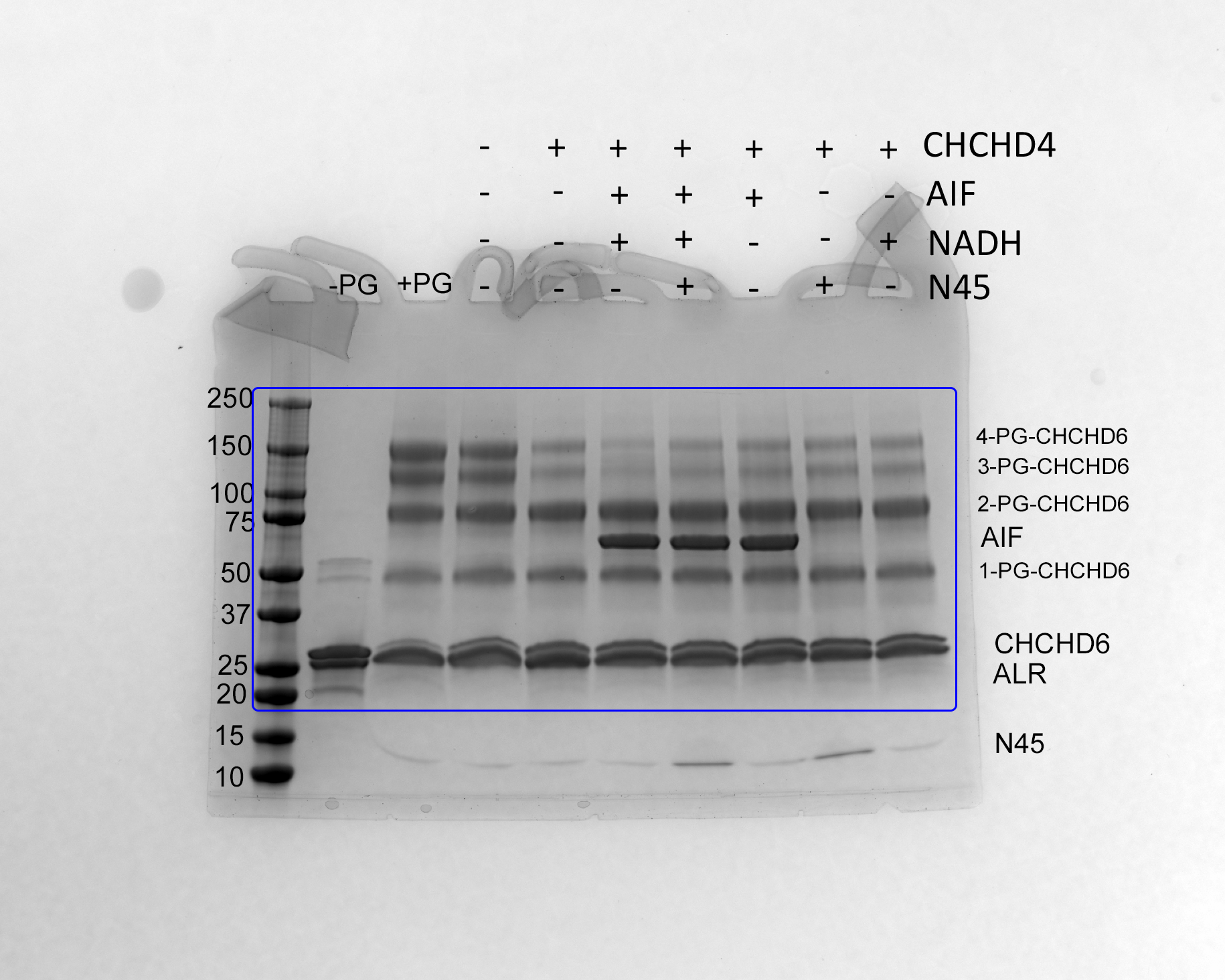

Supplement: Supplementary file 10 — Source data Fig. 6 [file 44318_2024_360_MOESM10_ESM.zip › Figure 6/6E/SDS-PAGE-AIF-CHCHD4-Refolding-15min.tif]

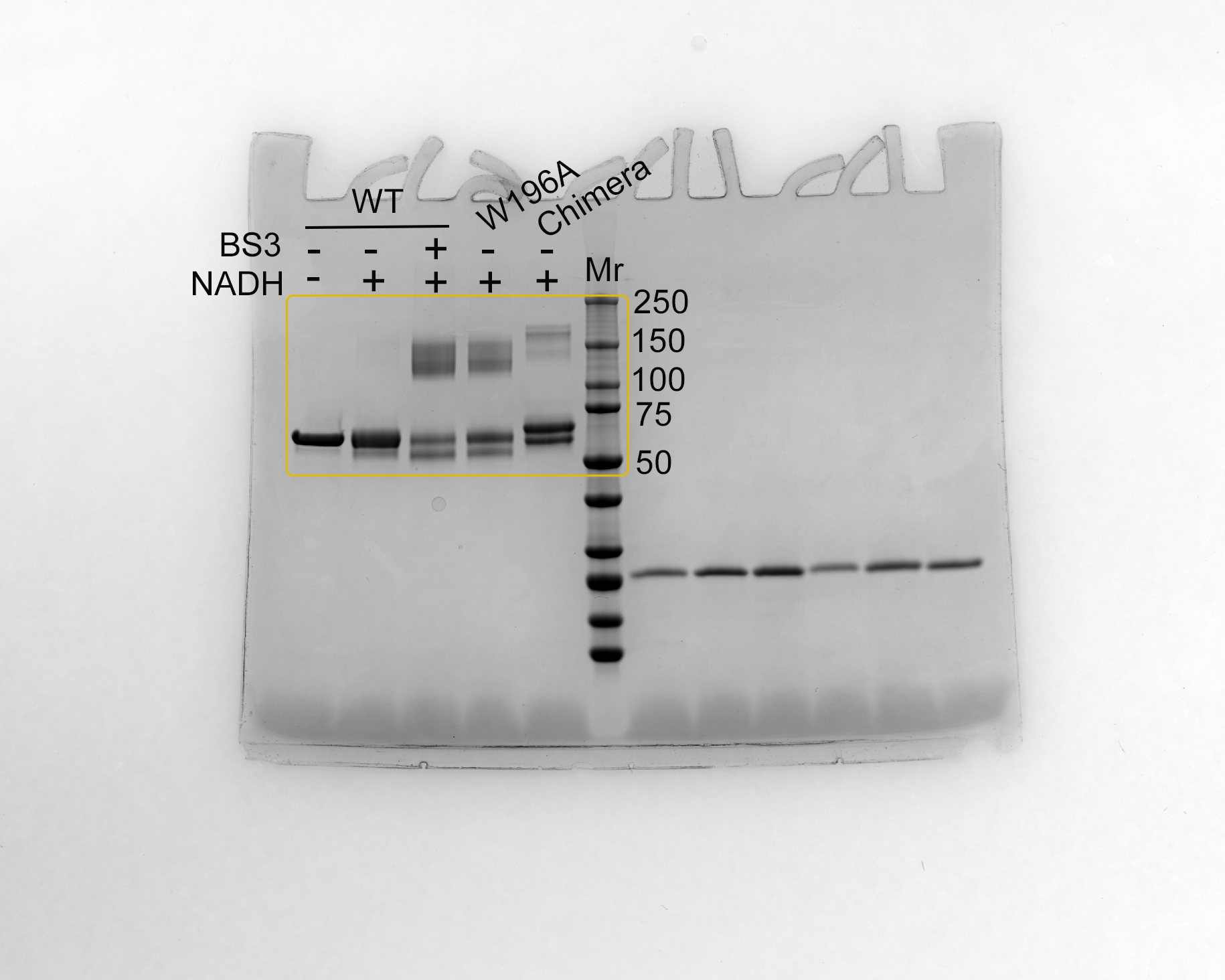

Supplement: Supplementary file 11 — Figure EV and Appendix Source Data [file 44318_2024_360_MOESM11_ESM.zip › Appendix/Appendix Figure 2/2A/2A-BS3-crosslinking/Appdendix-Figure-2A-SDS-PAGE-BS3.tif]

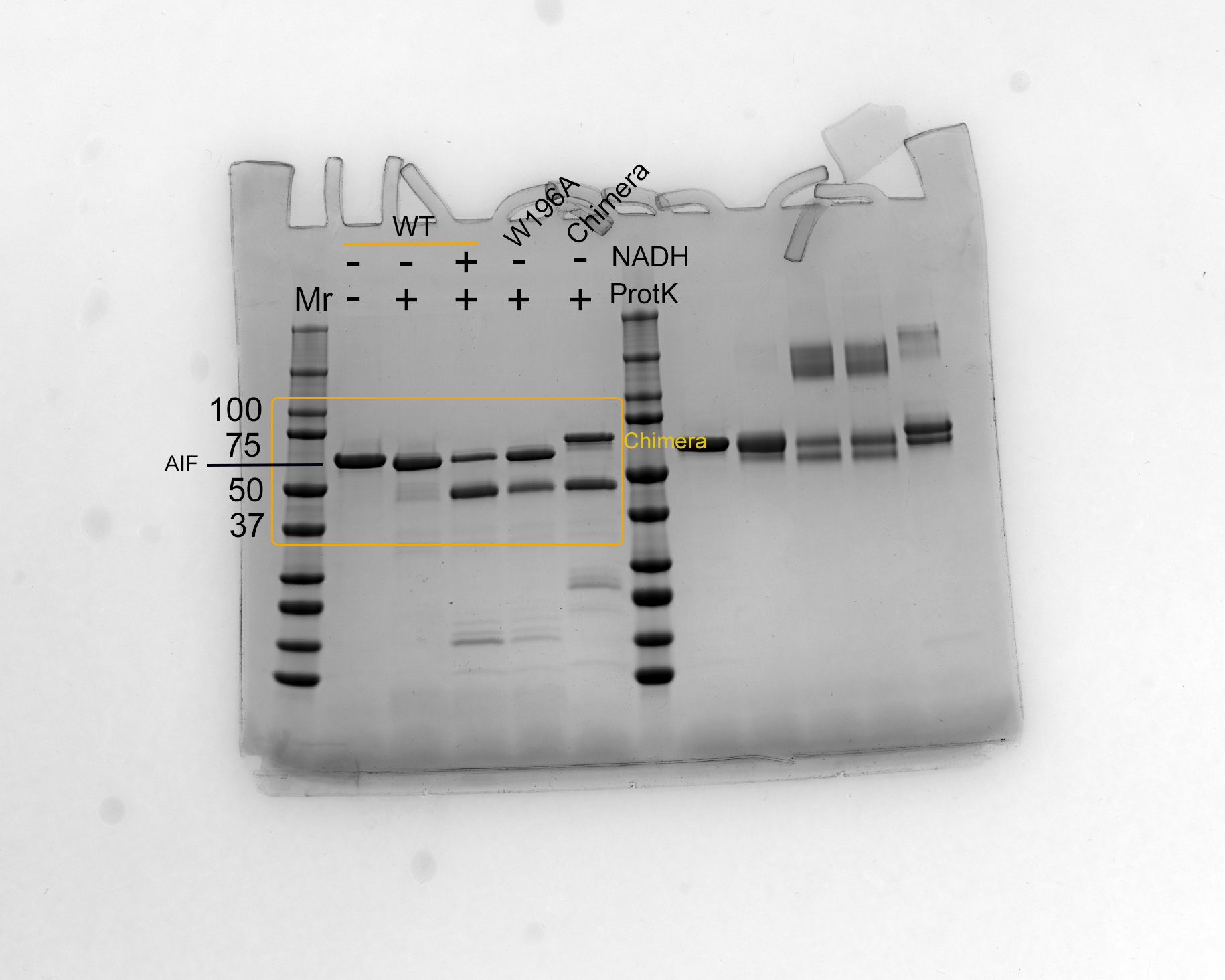

Supplement: Supplementary file 11 — Figure EV and Appendix Source Data [file 44318_2024_360_MOESM11_ESM.zip › Appendix/Appendix Figure 2/2A/2A-Limited-proteolysis/Appendix-Figure-2A-SDS-PAGE-LimProt.tif]

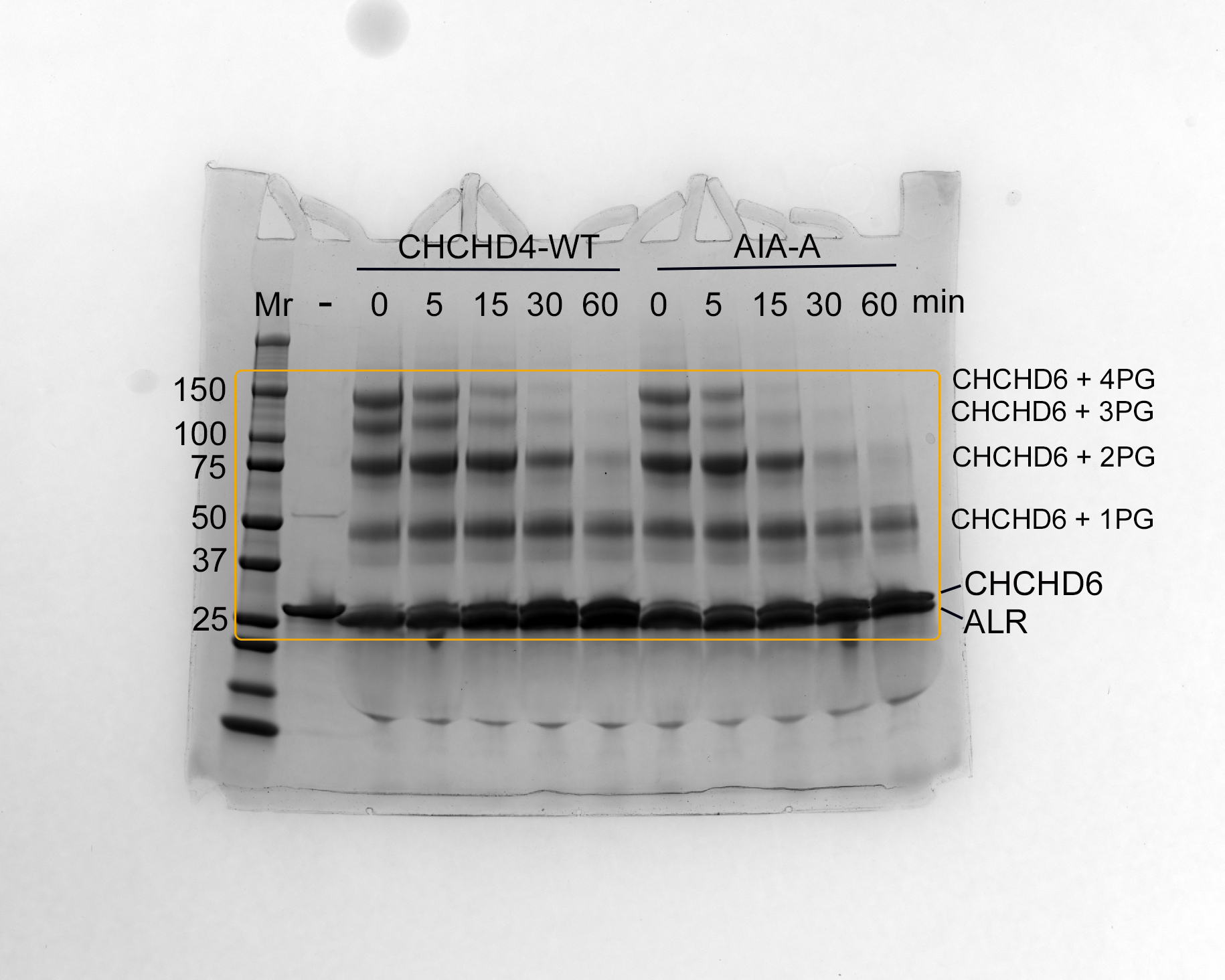

Supplement: Supplementary file 11 — Figure EV and Appendix Source Data [file 44318_2024_360_MOESM11_ESM.zip › Appendix/Appendix Figure 6/6A/Appendix-Figure-5A-SDS-PAGE.tif]

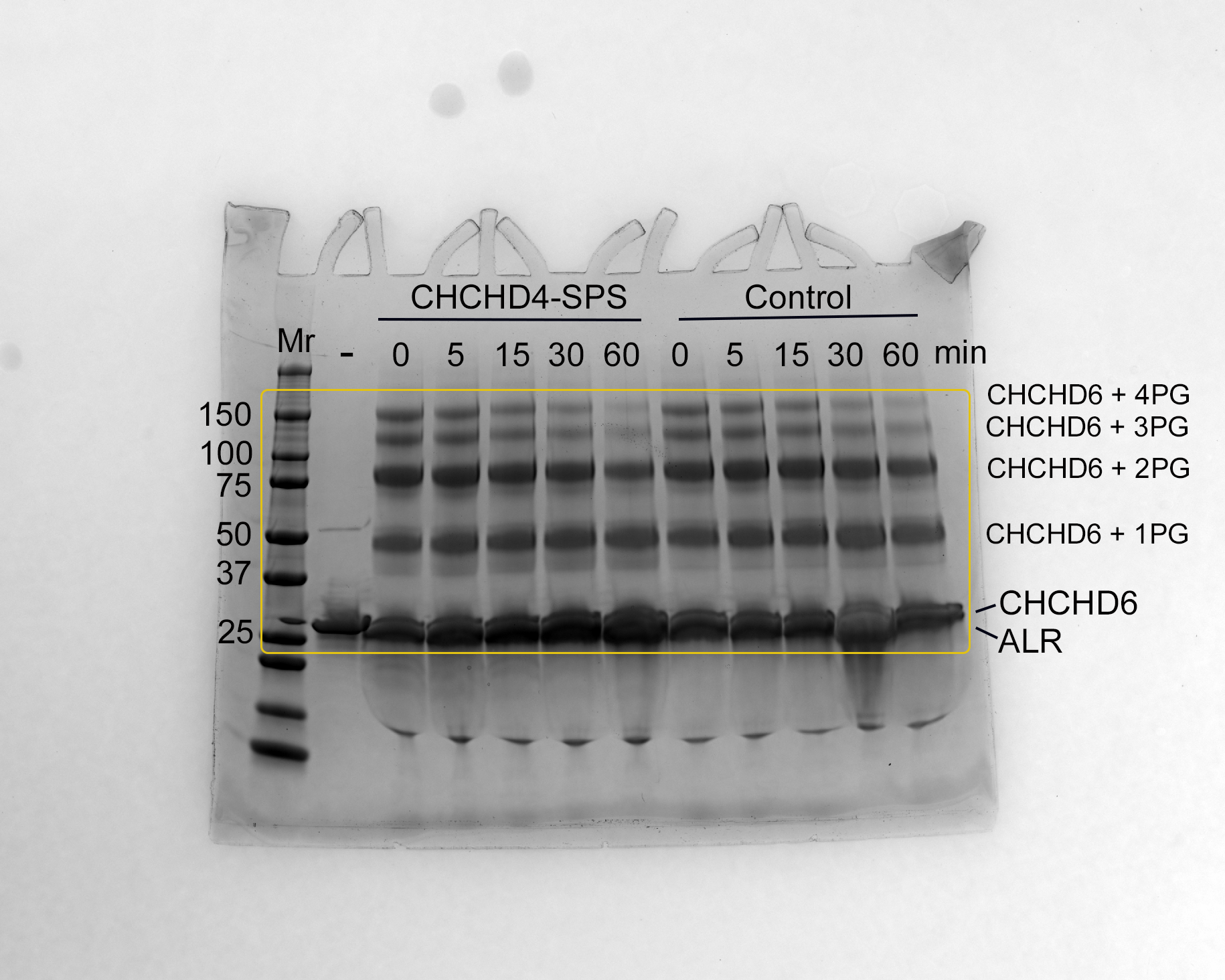

Supplement: Supplementary file 11 — Figure EV and Appendix Source Data [file 44318_2024_360_MOESM11_ESM.zip › Appendix/Appendix Figure 6/6B/Appendix-Figure-5B-SDS-PAGE.tif]

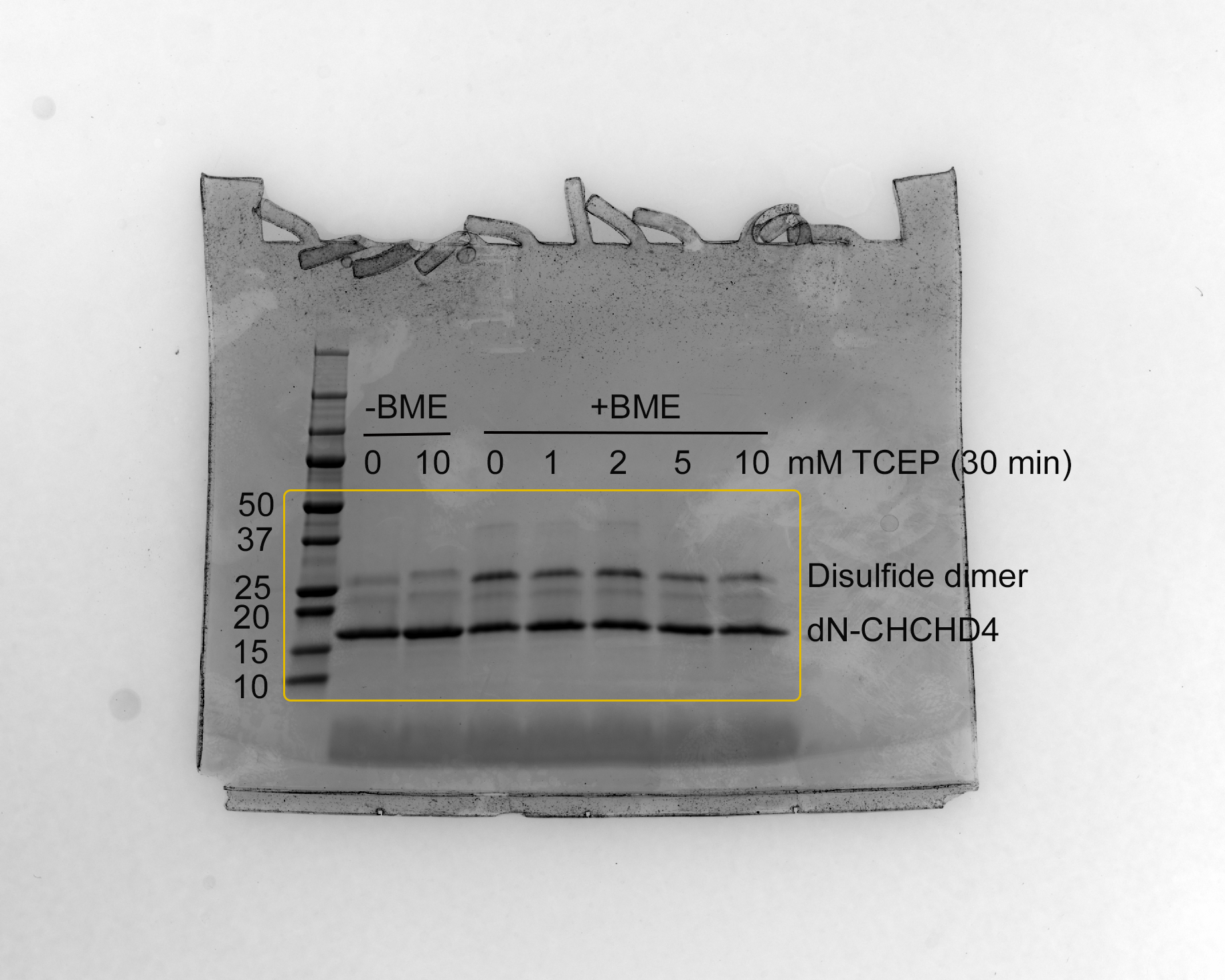

Supplement: Supplementary file 11 — Figure EV and Appendix Source Data [file 44318_2024_360_MOESM11_ESM.zip › Appendix/Appendix Figure 7/7E/Appendix-Figure-5G-SDS-PAGE.tif]

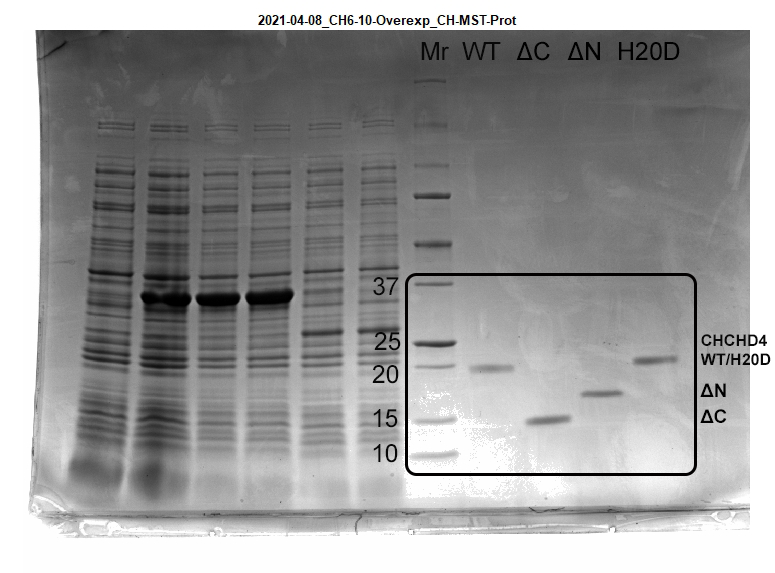

Supplement: Supplementary file 11 — Figure EV and Appendix Source Data [file 44318_2024_360_MOESM11_ESM.zip › Appendix/Appendix Figure 8/8A/Appendix-Fig-6A-SDS-PAGE-CHCHD4-Mutants-1.tif]

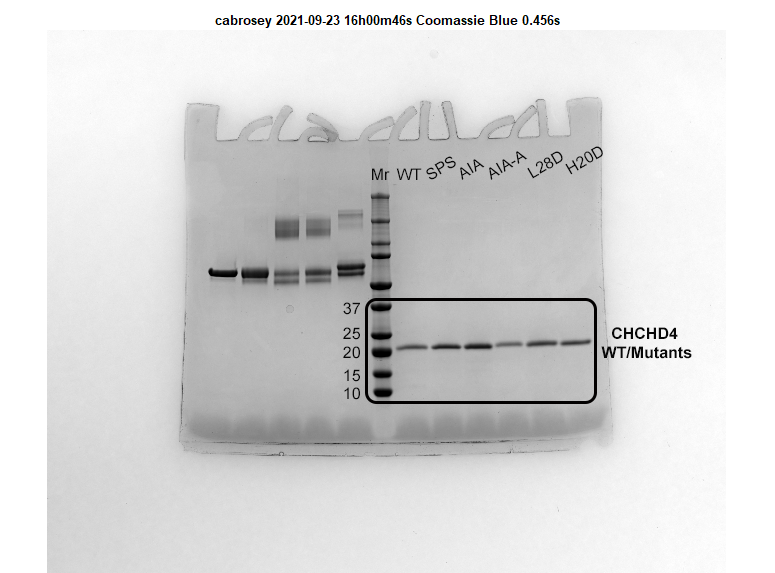

Supplement: Supplementary file 11 — Figure EV and Appendix Source Data [file 44318_2024_360_MOESM11_ESM.zip › Appendix/Appendix Figure 8/8A/Appendix-Fig-6A-SDS-PAGE-CHCHD4-Mutants-2.tif]

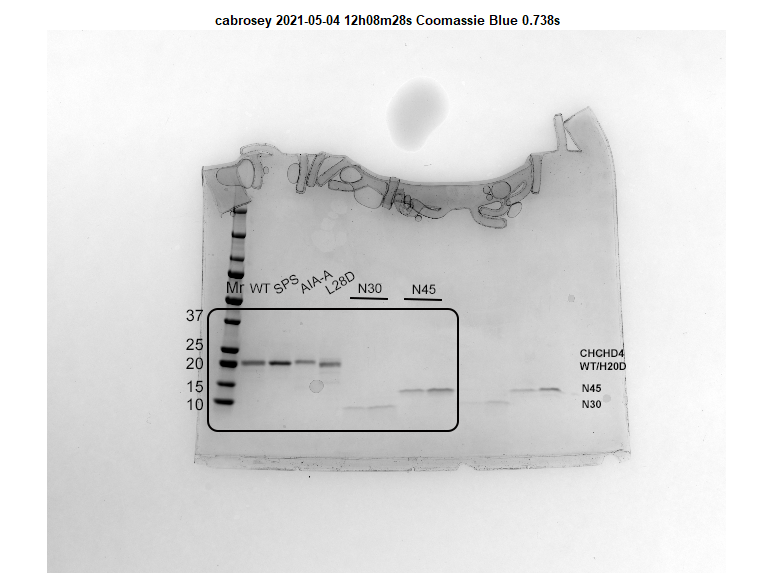

Supplement: Supplementary file 11 — Figure EV and Appendix Source Data [file 44318_2024_360_MOESM11_ESM.zip › Appendix/Appendix Figure 8/8A/Appendix-Fig-6A-SDS-PAGE-CHCHD4-Mutants-3.tif]

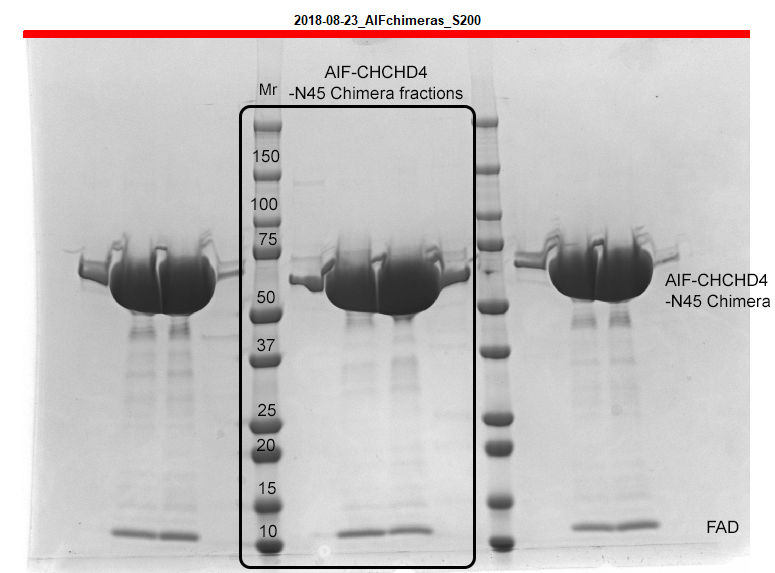

Supplement: Supplementary file 11 — Figure EV and Appendix Source Data [file 44318_2024_360_MOESM11_ESM.zip › Appendix/Appendix Figure 8/8B/Appendix-Fig-6B-AIF-CHCHD4-Chimera.tif]

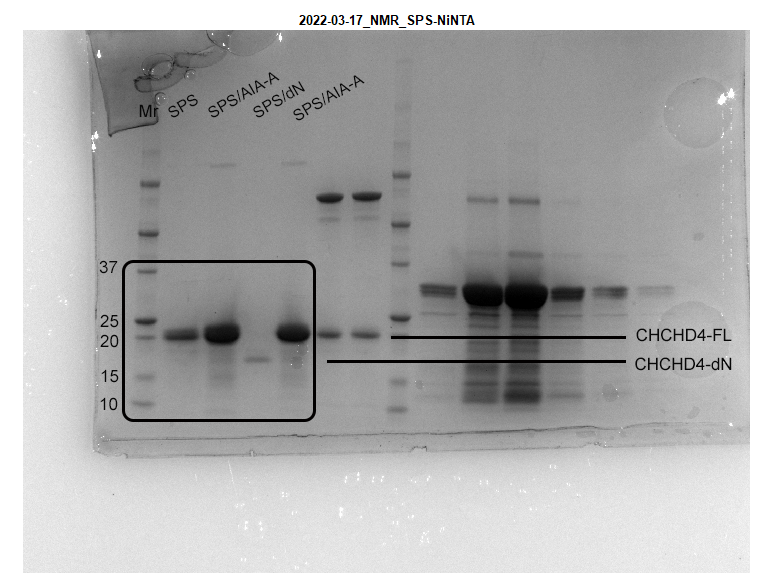

Supplement: Supplementary file 11 — Figure EV and Appendix Source Data [file 44318_2024_360_MOESM11_ESM.zip › Appendix/Appendix Figure 8/8C/Appendix-Fig-6C-NMR-Samples.tif]

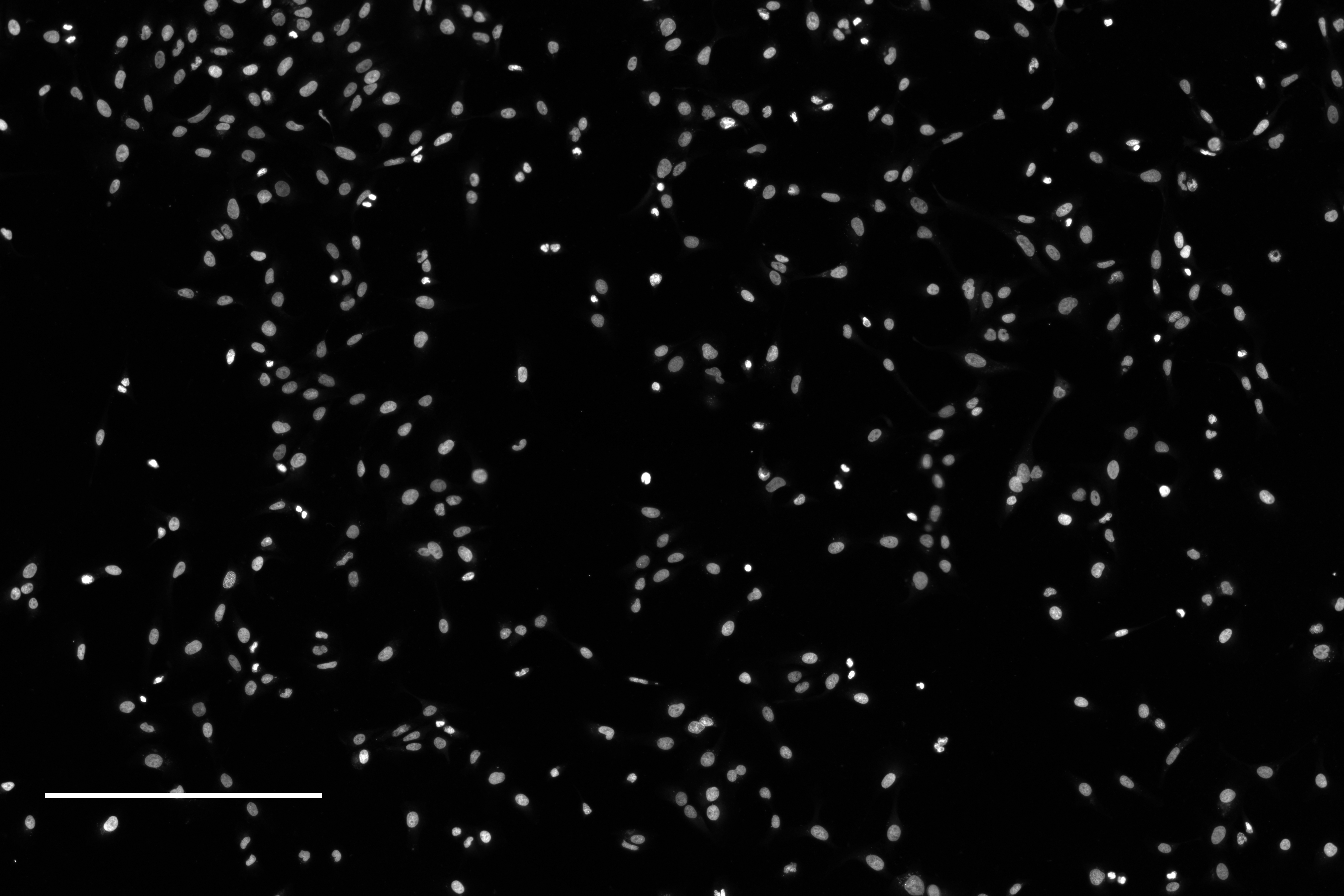

Supplement: Supplementary file 11 — Figure EV and Appendix Source Data [file 44318_2024_360_MOESM11_ESM.zip › Expanded-View/Figure 3/3A/N45-GFP-20X-DAPI.tif]

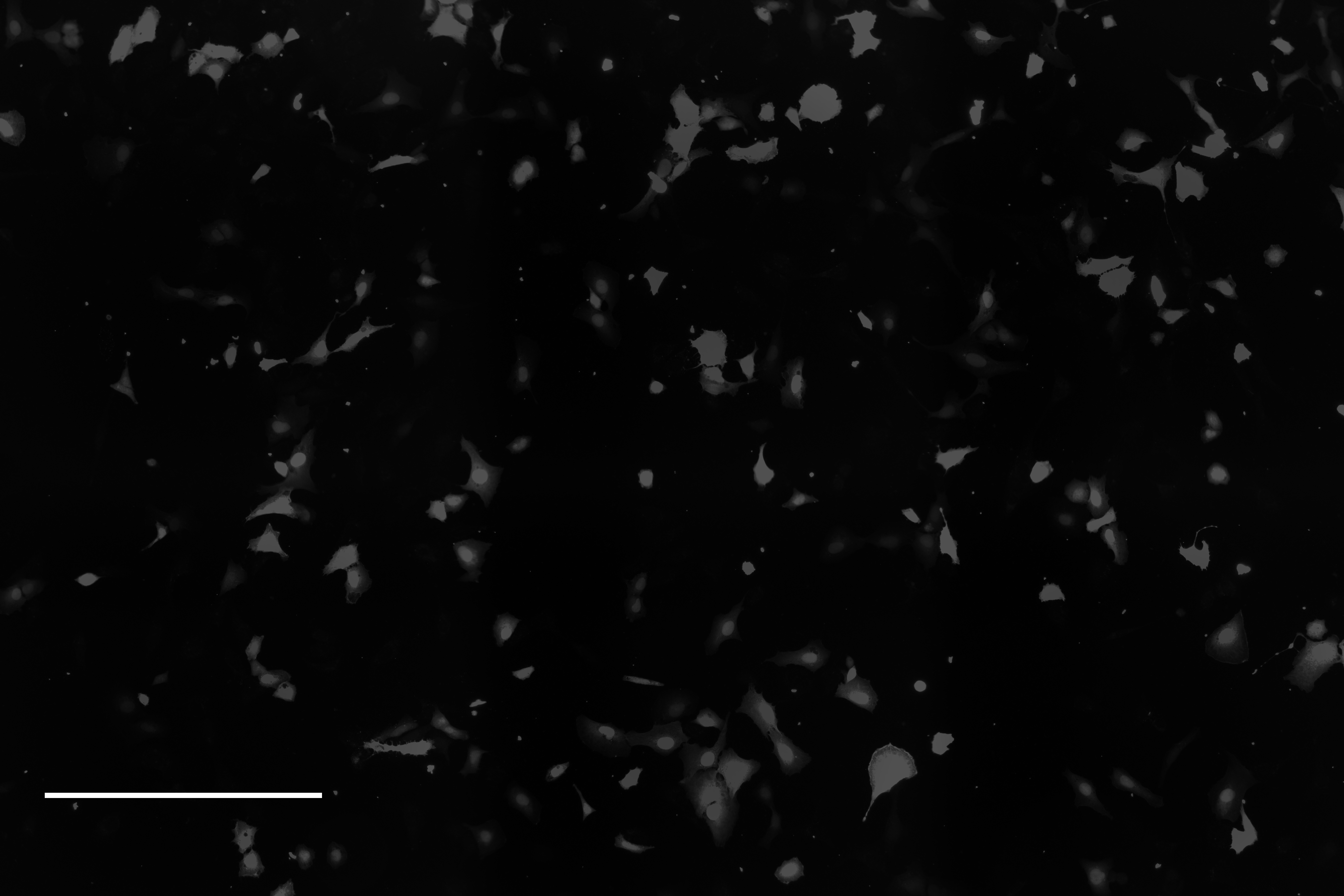

Supplement: Supplementary file 11 — Figure EV and Appendix Source Data [file 44318_2024_360_MOESM11_ESM.zip › Expanded-View/Figure 3/3A/N45-GFP-20X-GFP.tif]

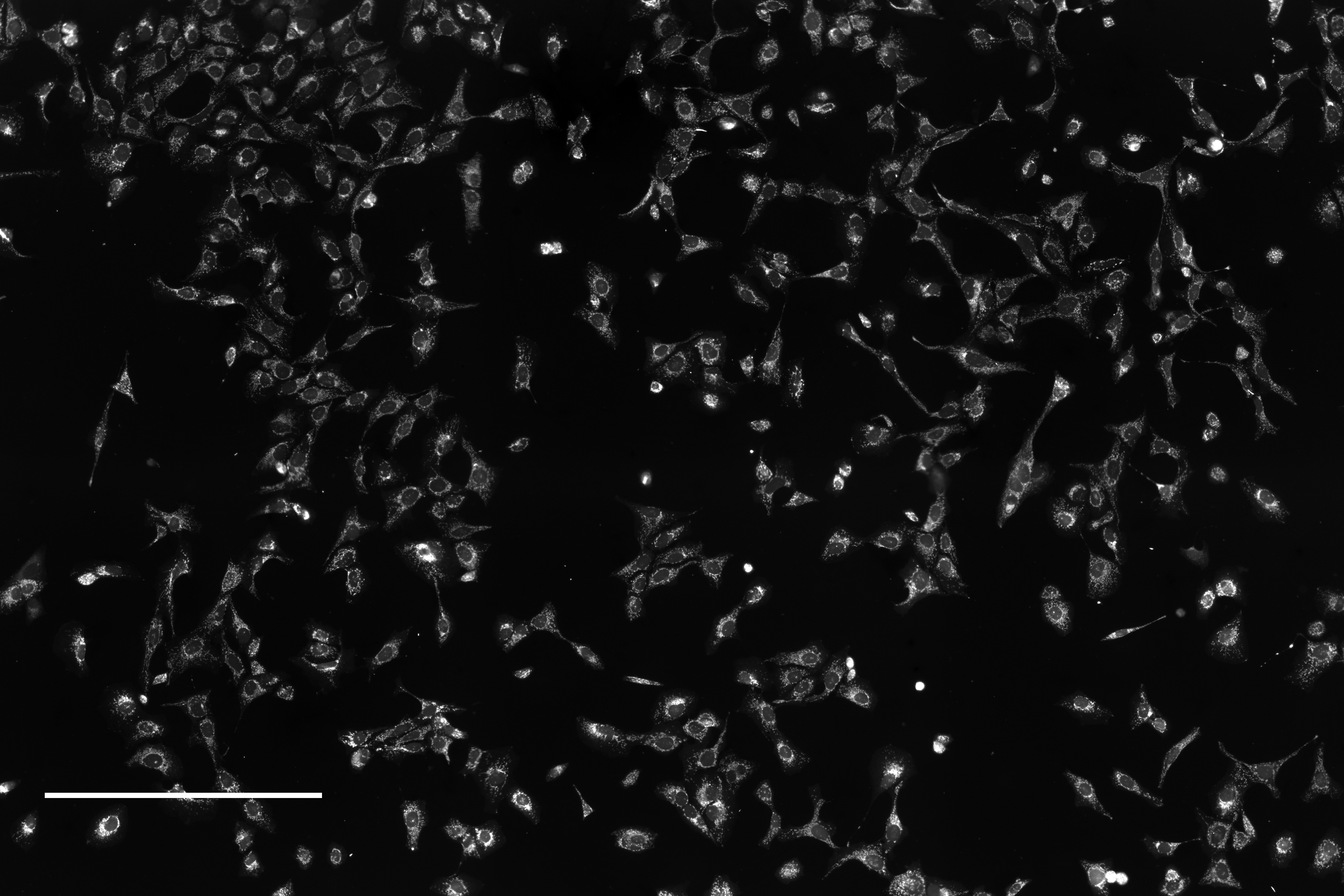

Supplement: Supplementary file 11 — Figure EV and Appendix Source Data [file 44318_2024_360_MOESM11_ESM.zip › Expanded-View/Figure 3/3A/N45-GFP-20X-Mitotracker.tif]
