# Supplementary material for: NADH-bound AIF activates the mitochondrial CHCHD4/MIA40 chaperone by a substrate-mimicry mechanism
Source: EMBO J. 2025 Jan 13;44(4):1220–48. doi: 10.1038/s44318-024-00360-6 (PMC11832770; doi:10.1038/s44318-024-00360-6)
Supplement: Supplementary file 12 — Expanded View Figures [file 44318_2024_360_MOESM12_ESM.pdf]

Expanded View Figures

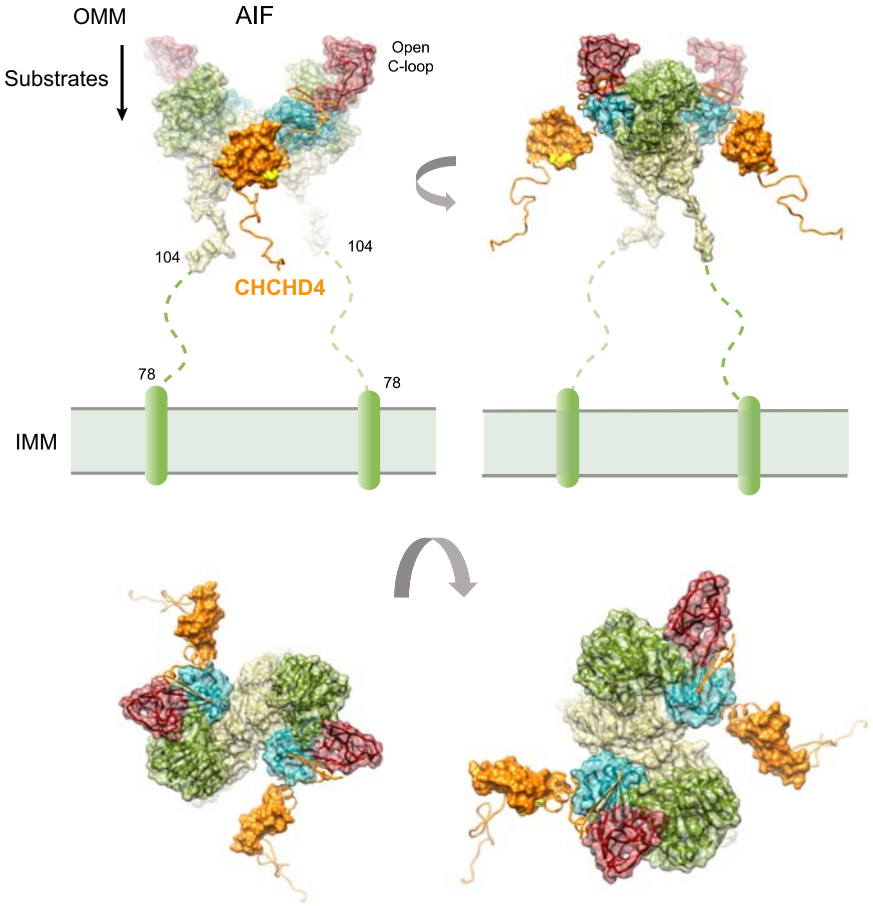

Figure EV1. Model of an AIF-CHCHD4 chaperone complex.

The crystal structure of the AIF-W196A-CHCHD4-N45 chimera and NMR structure of the catalytic CHCH domain (PDB: 2K3J) were used as templates to generate a model of the full-length complex by building flexible segments with the Modeller extension of Chimera. AIF residues 78–104 were left unmodeled for clarity. Top panels exhibit side views relative to the mitochondrial membranes, while lower panels view the complex overhead from the OMM.

 © The Author(s)

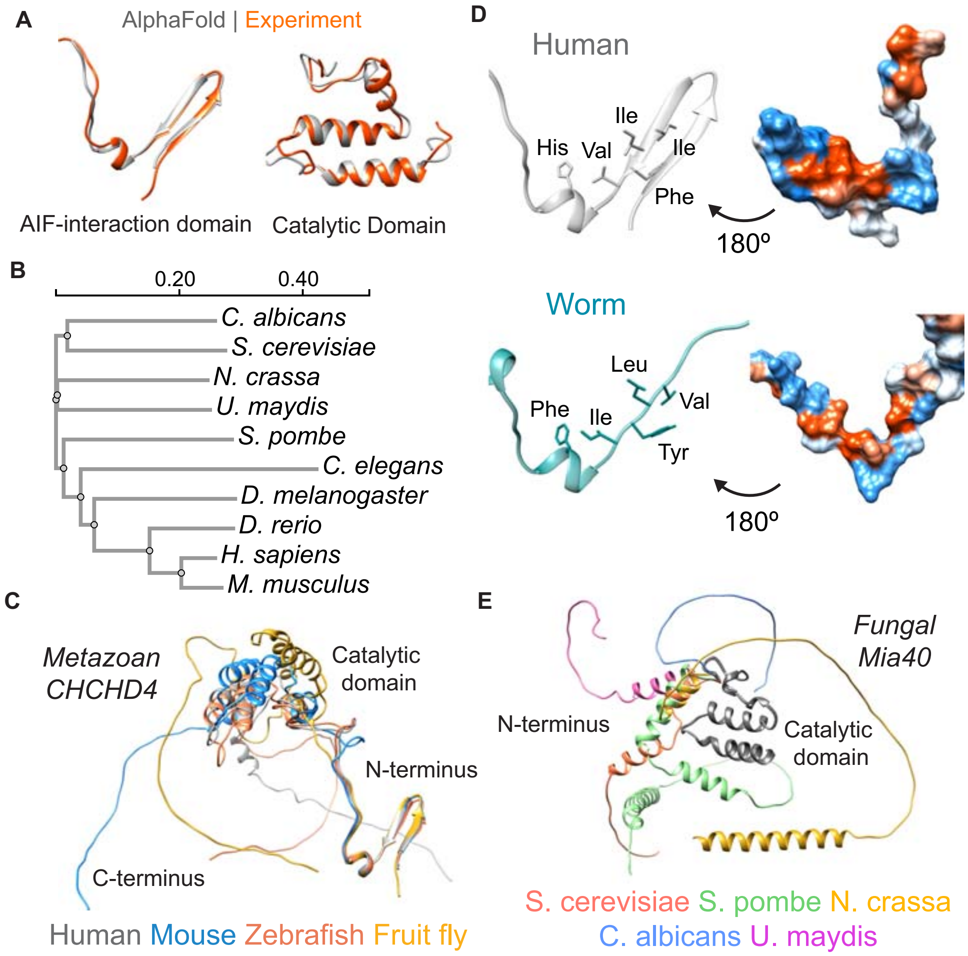

Figure EV2. CHCHD4's AIF-interaction motif is conserved across metazoan CHCHD4 homologs.

(**A**) Comparison of CHCHD4 experimental structures (orange) and AlphaFold models (gray) for the N-terminal AIF-interaction domain and central catalytic CHCH domain (PDB: 2K3J). (**B**) CHCHD4/Mia40 phylogenetic tree. Units indicate the average number of amino acid substitutions per site. (**C**) Superimposed AlphaFold models of metazoan CHCHD4 homologs. (**D**) The hydrophobic human AIF-interaction motif is conserved in *C. elegans* CHCHD4. Surfaces on right display Kyle–Doolittle hydrophobicity. (**E**) Superimposed AlphaFold models of fungal Mia40 homologs. C-terminal regions are excluded for clarity. Full-length models are displayed in Appendix Fig. S4.

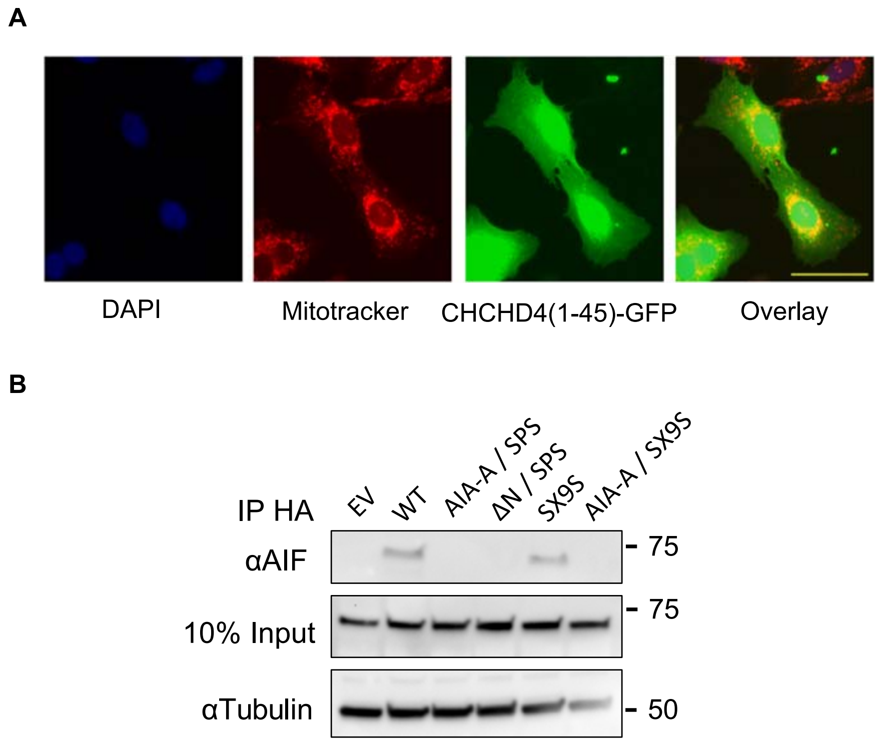

Figure EV3. CHCHD4's AIF-interaction domain is not an intrinsic mitochondrial localization sequence.

(**A**) CHCHD4 (1–45) fused to a C-terminal GFP tag was transiently expressed in U2-OS cells, which were co-labeled with Hoescht (DAPI) and Mitotracker dyes for visualization. Results are representative of three independent experiments. The scale bar is 50 microns. (**B**) Endogenous AIF is immunoprecipitated from 293T whole-cell lysates by HA-tagged CHCHD4 mutants containing wild-type AIF-interaction motifs. Results are representative of three independent experiments. Source data are available online for this figure.

 © The Author(s)

A

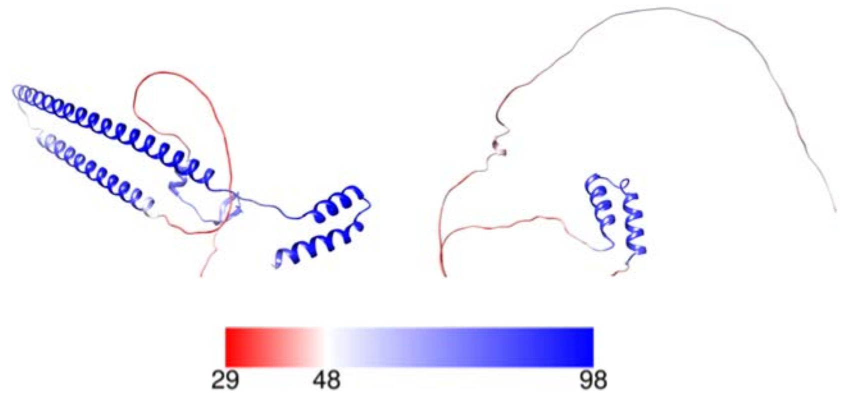

B

FL-SPS	ΔH (kcal/mol)	-TΔS (kcal/mol)	ΔG (kcal/mol)	Kd (μM)	N
CHCHD6	-5.77 (1.0)	-1.06 (1.17)	-6.13 (0.16)	33.2 (9.4)	1.17 (0.01)
CHCHD10	-11.2 (0.26)	4.83 (0.31)	-6.33 (0.18)	23.5 (7.5)	1.31 (0.12)
**ΔN-SPS**	ΔH (kcal/mol)	-TΔS (kcal/mol)	ΔG (kcal/mol)	Kd (μM)	N
CHCHD6	-9.32 (0.28)	2.76 (0.25)	6.57 (0.04)	15.5 (0.9)	1.00 (0.04)
CHCHD10	-18.63 (0.74)	11.57 (0.78)	-7.07 (0.02)	6.6 (0.2)	1.11 (0.05)
**AIAA-SPS**	ΔH (kcal/mol)	-TΔS (kcal/mol)	ΔG (kcal/mol)	Kd (μM)	N
CHCHD6	-4.78 (0.46)	-2.11 (0.40)	-6.89 (0.07)	9.0 (1.0)	1.16 (0.09)
CHCHD10	-15.17 (0.67)	7.86 (0.65)	-7.28 (0.04)	4.6 (0.3)	1.06 (0.06)

Figure EV4. The AIF-interaction motif regulates CHCHD4 substrate binding.

(**A**) AlphaFold models colored by pLDDT demonstrate high confidence in the helical hairpin structural prediction. (**B**) Summary of measured thermodynamic parameters from CHCHD4-substrate interactions. Values represent averages (standard deviations) from 2 to 3 independent experiments.

© The Author(s)
